# Unexpectedly Large Contribution of Oxygen to Charge Compensation Triggered by Structural Disordering: Detailed Experimental and Theoretical Study on a Li_3_NbO_4_–NiO Binary System

**DOI:** 10.1021/acscentsci.2c00238

**Published:** 2022-05-23

**Authors:** Ryutaro Fukuma, Maho Harada, Wenwen Zhao, Miho Sawamura, Yusuke Noda, Masanobu Nakayama, Masato Goto, Daisuke Kan, Yuichi Shimakawa, Masao Yonemura, Naohiro Ikeda, Ryuta Watanuki, Henrik L. Andersen, Anita M. D’Angelo, Neeraj Sharma, Jiwon Park, Hye Ryung Byon, Sayuri Fukuyama, Zhenji Han, Hitoshi Fukumitsu, Martin Schulz-Dobrick, Keisuke Yamanaka, Hirona Yamagishi, Toshiaki Ohta, Naoaki Yabuuchi

**Affiliations:** † Department of Applied Chemistry, Tokyo Denki University, 5 Senju Asahi-cho, Adachi-ku, Tokyo, Tokyo 120-8551, Japan; ‡ Frontier Research Institute for Materials Science (FRIMS), Nagoya Institute of Technology, Gokiso-cho, Showa-ku, Nagoya, Aichi 466-8555, Japan; § GREEN and MaDiS/CMi^2^, National Institute of Materials Science (NIMS), 1-2-1 Sengen, Tsukuba, Ibaraki 305-0047, Japan; ∥ Department of Information and Communication Engineering, Okayama Prefectural University, 111 Kuboki, Soja, Okayama 719-1197, Japan; ⊥ Elements Strategy Initiative for Catalysts and Batteries, Kyoto University, f1-30 Goryo-Ohara, Nishikyo-ku, Kyoto, Kyoto 615-8245, Japan; # Institute for Chemical Research, Kyoto University, Uji, Kyoto 611-0011, Japan; ∇ High Energy Accelerator Research Organization, Institute of Materials Structure Science, 1-1 Oho, Tsukuba, Ibaraki 305-0801, Japan; ○ Department of Materials Structure Science, The Graduate University for Advanced Studies, SOKENDAI, 203-1 Shirakata, Tokai, Ibaraki 319-1106, Japan; ◆ Department of Chemistry and Life Science, Yokohama National University, 79-5 Tokiwadai, Hodogaya-ku, Yokohama, Kanagawa 240-8501, Japan; ● School of Chemistry, University of New South Wales, Sydney, NSW 2052, Australia; ⧫ Australian Synchrotron, Clayton, VIC 3168, Australia; ◊ Department of Chemistry, KAIST Institute for NanoCentury, Korea Advanced Institute of Science and Technology (KAIST), 291 Daehak-ro, Yuseong-gu, Daejeon 34141, Republic of Korea; □ Battery Materials Laboratory, BASF Japan Ltd., 7-1-13 Doi-cho, Amagasaki, Hyogo 660-0083, Japan; ■ SR Center, Ritsumeikan University, 1-1-1 Noji-Higashi, Kusatsu, Shiga 525-8577, Japan; ⬠ Advanced Chemical Energy Research Center, Yokohama National University, 79-5 Tokiwadai, Hodogaya-ku, Yokohama, Kanagawa 240-8501, Japan

## Abstract

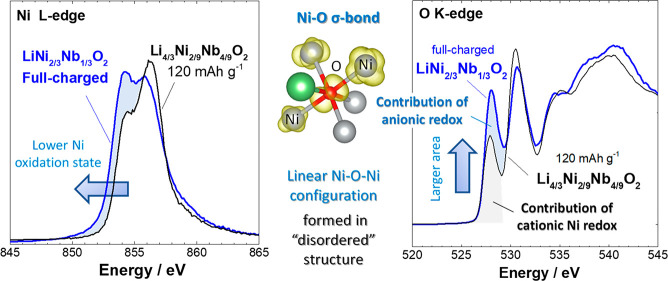

Dependence on lithium-ion batteries for automobile applications is rapidly increasing. The emerging use of anionic redox can boost the energy density of batteries, but the fundamental origin of anionic redox is still under debate. Moreover, to realize anionic redox, many reported electrode materials rely on manganese ions through π-type interactions with oxygen. Here, through a systematic experimental and theoretical study on a binary system of Li_3_NbO_4_–NiO, we demonstrate for the first time the unexpectedly large contribution of oxygen to charge compensation for electrochemical oxidation in Ni-based materials. In general, for Ni-based materials, *e.g*., LiNiO_2_, charge compensation is achieved mainly by Ni oxidation, with a lower contribution from oxygen. In contrast, for Li_3_NbO_4_–NiO, oxygen-based charge compensation is triggered by structural disordering and σ-type interactions with nickel ions, which are associated with a unique environment for oxygen, *i.e*., a linear Ni–O–Ni configuration in the disordered system. Reversible anionic redox with a small hysteretic behavior was achieved for LiNi_2/3_Nb_1/3_O_2_ with a cation-disordered Li/Ni arrangement. Further Li enrichment in the structure destabilizes anionic redox and leads to irreversible oxygen loss due to the disappearance of the linear Ni–O–Ni configuration and the formation of unstable Ni ions with high oxidation states. On the basis of these results, we discuss the possibility of using σ-type interactions for anionic redox to design advanced electrode materials for high-energy lithium-ion batteries.

## Abstract 2

Reversible anionic redox with σ-type interactions, which is used by high-energy electrode materials for battery applications, is first realized for Ni-based materials through structural disordering with Nb ions.

## Introduction

With three decades of history, lithium-ion battery (LIB) research and development is highly sophisticated,^1^ with Ni-based layered materials^2−4^ currently dominating the market for positive electrode materials. Currently, the lack of next-generation positive electrode materials, which practically outperform the Ni-based layered materials, hinders the further development of LIBs. Over the past decade, Li-excess manganese oxide, Li_2_MnO_3_, and its derivatives have been intensively studied as potential next-generation positive electrode materials for LIBs.^5−7^ Anomalously large reversible capacities have been observed for these electrode materials^8−11^ compared with classical and stoichiometric layered materials in which charge compensation during electrochemical Li extraction is achieved by reversible redox reactions of transition metal ions as cationic species. Significant research effort has revealed that charge compensation proceeds in both cationic and anionic species.^12,13^ In a model system, Li_2_Ru_1–*x*_Sn_*x*_O_2_, the origin of the large reversible capacity (∼260 mAhg^–1^) was proposed to be the reversible formation of oxo- to peroxo-like species, namely anionic redox.^14^ It has also been proposed that charge compensation is achieved by the formation of localized electron holes on oxygen atoms coordinated by Li^+^ and Mn^4+^.^15,16^ A recent finding also suggests that the oxidation of oxygen results in the formation of oxygen molecules trapped in oxides, which are reduced back to oxide ions via reduction.^17^ Nevertheless, for the Li_2_MnO_3_-based system, oxygen loss during the initial charge process and voltage decay during cycling are inevitable.^18^ Oxygen loss activates Mn^3+^/Mn^4+^ and Co^2+^/Co^3+^ redox during electrochemical cycles, leading to a lower operating voltage.^19^ The article morphology,^20^ the surface coating,^21^ the selection of a binder,^22^*etc*. influence voltage fading, but its suppression is a kinetically controlled phenomena. Relatively large voltage hysteresis for the charge–discharge process has also been noted.^23^ Voltage hysteresis is influenced by in-plane cation ordering^17^ and electron delocalization associated with the stabilization holes and the suppression of oxygen dimers.^24,25^ Such disadvantages hinder the use of these high-capacity next-generation materials for practical applications. Other Li-excess systems, including Li_4_MoO_5_, Li_3_NbO_4_, and Li_2_TiO_3_, have been also proposed as host structures for anionic redox.^26−28^ Recently, the concept of anionic redox was extended to the Li-excess metal oxyfluorides with the rocksalt structure.^29−34^ Oxygen loss during electrochemical cycles is partially mitigated by fluorination because of the increase in the contribution of Mn cationic redox.^35^ Anionic redox has been evidenced not only for rocksalt-related structures but also for antifluorite-related structures^36−39^ and metal silicates.^40^

Such oxygen redox chemistry potentially opens a new path to the design of energy storage materials, but many questions still remain unanswered. Theoretical studies of the Li-excess system have suggested that oxygen redox is activated in O 2p orbitals, which interact with excess Li ions in the transition metal layer (namely, oxygen redox occurs in compounds that contain linear Li^+^–O–Li^+^ environments).^16,41^ Recently, this concept was extended to Mg^2+^.^42^ Ions with a highly ionic character donate more electrons to the oxygen O 2p orbital, thus the energy level of the O 2p orbital approaches the Fermi level. Therefore, oxygen redox is easily accessed at a lower electrochemical potential for compounds containing ions with a highly ionic bonding nature toward oxide ions, such as Mg^2+^, Ti^4+^, Nb^5+^, *etc*.^43^ Nevertheless, as pointed out in a recent article,^44^ the nature of the chemical bonds between oxygen and the ions without valence electrons is essentially a nonbonding state, therefore these ions cannot stabilize the redox reaction of oxide ions. Oxygen redox chemistry was proposed to be energetically stabilized through π-type electron donation from Mn t_2g_ orbitals.^25,45^ Therefore, the enrichment of ions with a d^0^ configuration and an ionic bonding nature (*e.g*., Nb^5+^ and Ti^4+^) in the structure leads to the reduction in the number of oxide ions coordinated to transition metal ions (*e.g*., Mn), which can stabilize anionic redox. To access oxygen redox at reasonable potentials (within the current electrolyte window), ions with d^0^ configurations are critical; however, to ensure the reversibility of the redox process or stabilize the intermediate oxygen-based species, electron donation from metals such as Mn and Ni (those with d electrons) are critical. These two apparently opposing factors cause a dilemma regarding the design of materials with anionic redox for rechargeable battery applications. This provides opportunity for a careful materials design strategy that incorporates both d^0^ and d^*n*^ transition metals in compounds.

In this work, detailed systematic studies are conducted on a binary system of Li_3_NbO_4_–NiO for lithium storage applications, taking full advantage of the anionic redox reaction. In our preliminary study on Li_1.3_Nb_0.43_Ni_0.27_O_2_, which is found in this binary system, its crystal structure and the electrochemical properties were reported.^46^ Anomalously large voltage hysteresis during charge–discharge was noted and compared with Li_1.3_Nb_0.3_Mn_0.4_O_2_, suggesting that anionic redox is less stabilized for Li_1.3_Nb_0.43_Ni_0.27_O_2_. This work is a systematic study of this binary system, a Mn-free system. We report for the first time a significant the contribution of oxygen to the charge compensation mechanism. The formation of an oxygen unoccupied state was observed in stoichiometric LiMeO_2_ (Me = Ni^2+^ and Nb^5+^), and charge compensation by oxygen was unexpectedly large compared with that by nickel ions as cationic species. For conventional layered electrode materials with Ni ions, such as LiNiO_2_ and LiNi_1/2_Mn_1/2_O_2_, charge compensation is achieved mainly by Ni oxidation upon delithiation, and the contribution from oxygen is relatively small. The proposed process in the Ni or Nb system obviously differs from these conventional materials and is classified as anionic redox, where the oxidation reaction of oxygen is stabilized by a σ-type interaction with the Ni e_g_ orbital. Moreover, oxygen is reduced with a relatively small voltage hysteresis upon discharge (surprisingly less than 0.3 V, a value significantly smaller than that observed for the Mn system^47^). In contrast, for the Li-excess composition, Li_1.33_Me_0.67_O_2_, oxygen loss is dominant as an irreversible process. These results indicate that anionic redox is energetically stabilized for the stoichiometric composition and Li enrichment in the structure results in destabilization. This trend is clearly different from the activation and stabilization processes of anionic redox in the Mn system that rely on the interaction between oxygen 2p and Mn t_2g_ orbitals, and this finding contributes to a better understanding of anionic redox with a different chemistry related to the oxygen coordination. From these results, we discuss the possibility of highly reversible anionic redox in the 4 V class of electrode materials.

## Results and Discussion

### Structural and Morphological Characterization of the Li_3_NbO_4_–NiO Binary System

**Figure 1:**
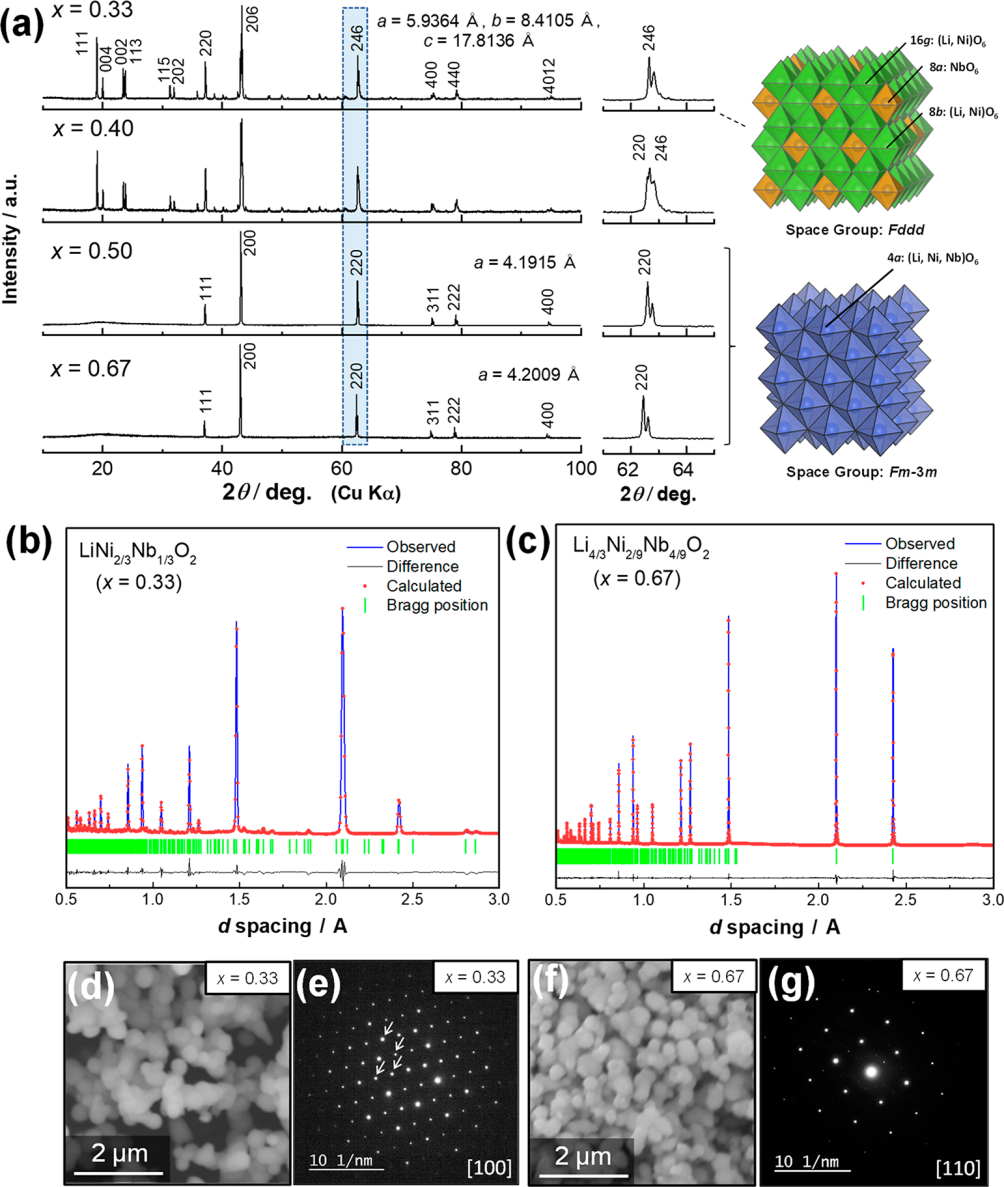
Structural and morphological characterization of the *x*Li_3_NbO_4_–(1 – *x*)NiO binary phase. (a) XRD patterns and schematic illustrations of the crystal structures. Enlarged patterns are also shown. Rietveld fits of structural models to neutron diffraction data for (b) LiNi_2/3_Nb_1/3_O_2_ (*x* = 0.33) and (c) Li_4/3_Ni_2/9_Nb_4/9_O_2_ (*x* = 0.67). The samples used for the Rietveld analysis were synthesized at 1000 °C for 48 h. Peak splitting for the samples where *x* = 0.5 and 0.67 originates from a non-monochromatic X-ray source. SEM and ED patterns, respectively, of (d and e) LiNi_2/3_Nb_1/3_O_2_ (*x* = 0.33) and (f and g) Li_4/3_Ni_2/9_Nb_4/9_O_2_ (*x* = 0.67). The samples used for The SEM and ED measurements were synthesized at 1000 °C for 2 h (also see Supporting Figure S6). Arrows in panel e correspond to superlattice reflections based on a parent rocksalt phase. Schematic illustrations of crystal structures were drawn using VESTA.^78^

The crystal structures of the as-prepared samples were analyzed by both X-ray diffraction (XRD) and neutron diffraction (ND). Powder XRD patterns of the binary system of *x*Li_3_NbO_4_–(1 – *x*)NiO are shown in Figure 1. For the Li_3_NbO_4_-rich phases Li_4/3_Ni_2/9_Nb_4/9_O_2_ (*x* = 0.67) and Li_6/5_Ni_2/5_Nb_2/5_O_2_ (*x* = 0.50), diffraction lines were assigned as a cation-disordered rocksalt-type structure with a cubic symmetry (*Fm*3̅*m* space group). For the NiO-rich phase (LiNi_2/3_Nb_1/3_O_2_, *x* = 0.33), major diffraction lines were also assigned as the rocksalt phase, but some additional diffraction lines were also observed in the 2θ range of 20–35°. All diffraction lines were successfully indexed to an orthorhombic lattice with a *Fddd* space group. These additional lines originate from Nb ordering at the octahedral sites of the rocksalt phase, and we note that the Nb ordering is within a common close-packed cubic lattice of oxide ions. The positions of the fundamental diffraction lines for the rocksalt phase gradually shift to higher diffraction angles as *x* decreases from 0.67 to 0.33, which is clearly visible in the highlighted 220 diffraction line in the 2θ range of 62–64°. Because the ionic radius of the Ni^2+^ ion (0.83 Å) is smaller than that of the Li^+^ ions (0.90 Å) in an octahedral environment,^48^ lattice parameters and the unit cell volume (in the rocksalt structure) gradually shrink as the NiO fraction increases. Detailed crystal structures of stoichiometric LiNi_2/3_Nb_1/3_O_2_ and Li-excess Li_4/3_Ni_2/9_Nb_4/9_O_2_ were refined by the Rietveld analysis of structural models using both XRD and ND patterns. Results obtained from the ND data are shown in Figure 1b and c and the corresponding refined structural parameters are summarized in Supporting Tables S1 and S2, while the results derived from the XRD data are shown in Supporting Figure S1a and b and corresponding the structural parameters are also shown in Supporting Tables S1 and S2. The crystal structure of LiNi_2/3_Nb_1/3_O_2_ is also consistent with the data reported in the literature.^49^ Perfect Nb ordering is evidenced, while Ni and Li ions are essentially disordered in this structure. For Li_12/11_Ni_6/11_Nb_4/11_O_2_ (*x* = 0.40), broad diffraction lines suggest phase segregation, as shown in highlighted diffraction patterns of Figure 1a, and the sample consists of both *Fddd* and *Fm*3̅*m* phases. Mass fractions by calculated by Rietveld analysis to be 60% for the *Fddd* phase and 40% for the *Fm*3̅*m* phase. A single-phase sample was not obtained by applying the experimental conditions used in this study.

The particle morphology was further examined using SEM and TEM measurements. From SEM images of the stoichiometric and Li-excess samples, it was noted that the as-prepared samples had almost identical particle morphologies and particle sizes (∼200 nm), as shown in Figure 1d and f. Spherical particles with a uniform size around 200 nm were observed for both samples. The difference in the chemical composition does not affect the particle morphology of the as-prepared samples in this system (also see Supporting Figure S2). Spherical particles with a smooth surface are also noted from TEM examinations, as shown in Supporting Figure S3. The particles are interconnected, which is clearly evidenced by the presence of grain boundaries. The selected area electron diffraction (SAED) patterns of stoichiometric LiNi_2/3_Nb_1/3_O_2_ (Figure 1e) and Li-excess Li_4/3_Ni_2/9_Nb_4/9_O_2_ (Figure 1g) samples collected from the same zone axis on the base of a primitive rocksalt cell clearly indicatethea highly crystalline nature of both samples with well-defined diffraction spots. For the stoichiometric phase, both clear super-lattice reflections and fundamental rocksalt spots were observed, which is consistent with the results of Rietveld analysis associated with the Nb ordering. Further analysis, including the cation ordering of Nb ions in LiNi_2/3_Nb_1/3_O_2_, of the SAED patterns is described in Supporting Figures S4 and S5. Note that a binary system of Li_3_NbO_4_–(1 – *x*)CoO shows crystal structures and particle morphologies (under preparation) similar to those of the Ni system. This fact indicates that the enrichment of the Li_3_NbO_4_ fraction disturbs the cation ordering in the cubic-close-packed array of oxide ions, energetically stabilizing the disordered rocksalt structure.

### Electrochemical Properties within the Li_3_NbO_4_–NiO Binary System

**Figure 2:**
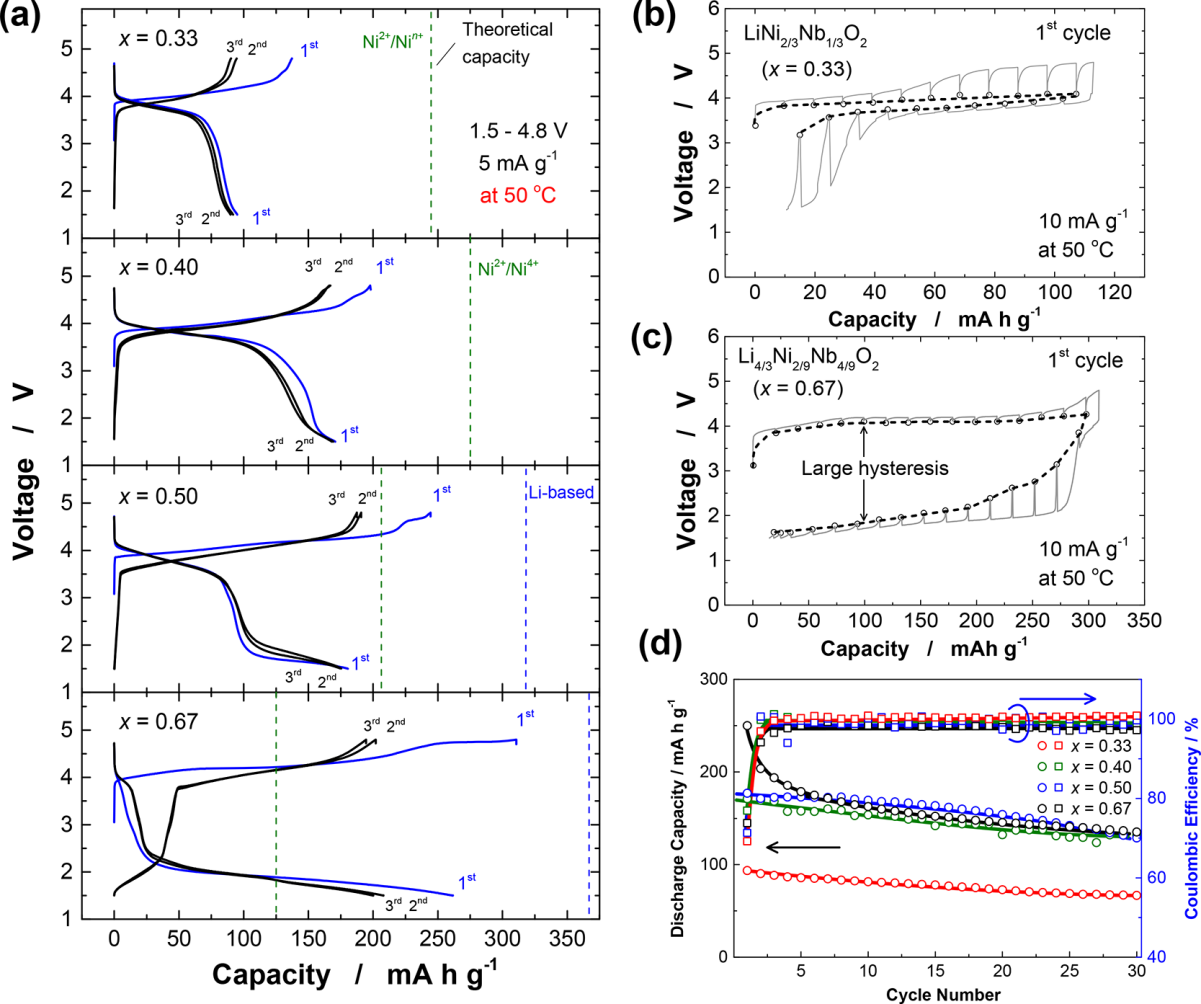
Electrochemical properties of *x*Li_3_NbO_4_ – (1 – *x*) NiO binary phases in Li cells. The samples used herein were synthesized at 1000 °C for 2 h. (a) Charge–discharge profiles of Li–Ni–Nb–O samples cycled between 1.5 and 4.8 V at a specific current of 5 mA g^–1^. Galvanostatic intermittent titration curves of (b) LiNi_2/3_Nb_1/3_O_2_ (*x* = 0.33) and (c) Li_4/3_Ni_2/9_Nb_4/9_O_2_ (*x* = 0.67). The cells were charged for 1 and 2 h in LiNi_2/3_Nb_1/3_O_2_ and Li_4/3_Ni_2/9_Nb_4/9_O_2_, respectively, then rested for 4 h. (d) Capacity retention and Coulombic efficiency of the binary system at a specific current of 5 mA g^–1^.

Electrode performance of the binary phase in Li cells was examined using galvanostatic charge–discharge at 50 °C, and the results are summarized in Figure 2. Although the samples synthesized for a longer time (48 h) have higher degrees of cation ordering, their larger particle sizes result in poor electrode performance. as shown in Supporting Figure S6. Therefore, the electrode performance was examined for the samples synthesized at 1000 °C for 2 h. For stoichiometric LiNi_2/3_Nb_1/3_O_2_, approximately 100 mA h g^–1^ of reversible capacity was observed at a rate of 5 mA g^–1^ at 50 °C, with an average redox potential of ∼4 V and small polarization and voltage hysteresis. The small voltage hysteresis is further evidenced by the measurement of the quasi-open-circuit voltage (Figure 2b). The redox reaction for the stoichiometric phase is highly reversible, and the operating voltage reaches nearly 4 V. Nevertheless, the observed reversible capacity is smaller than the theoretical capacity (245 mA h g^–1^), which is further discussed in a later section. In addition, the observed reversible capacity decreased to 60 mA h g^–1^ at a rate of 20 mA g^–1^ (Supporting Figure S7a), indicating that the stoichiometric sample had inferior electrode kinetics. Further increasing the fraction of Li_3_NbO_4_ led to an increase in the reversible capacity. Li-excess Li_4/3_Ni_2/9_Nb_4/9_O_2_ delivers a large initial charge capacity, over 300 mA h g^–1^, which significantly exceeds the theoretical expectation on the basis of Ni^2+^/Ni^4+^ redox. This observation suggests that oxide ions are involved in the charge compensation process. Nevertheless, a large polarization with a capacity of 250 mA h g^–1^ was evidenced upon discharge. In addition, the poor reversibility of the electrode materials was observed, as shown in Figure 2d. To further examine the electrode reversibility of Li_4/3_Ni_2/9_Nb_4/9_O_2_, the initial charge capacities were changed from 100 to 300 mA h g^–1^, followed by discharge to 1.5 V (Supporting Figure S7b). When the charge capacity was limited to 100 mA h g^–1^, better reversibility was observed. As the charge capacity increased, the polarization upon discharge gradually increased, as can be seen from the appearance of a discharge plateau at 1.5–2.0 V. Notably, large hysteresis was still observed in the open-circuit voltage measurements (Figure 2c), which will also be discussed in a later section.

### Reaction Mechanisms of Samples in the Li_3_NbO_4_–NiO Binary System in Li Cells

**Figure 3:**
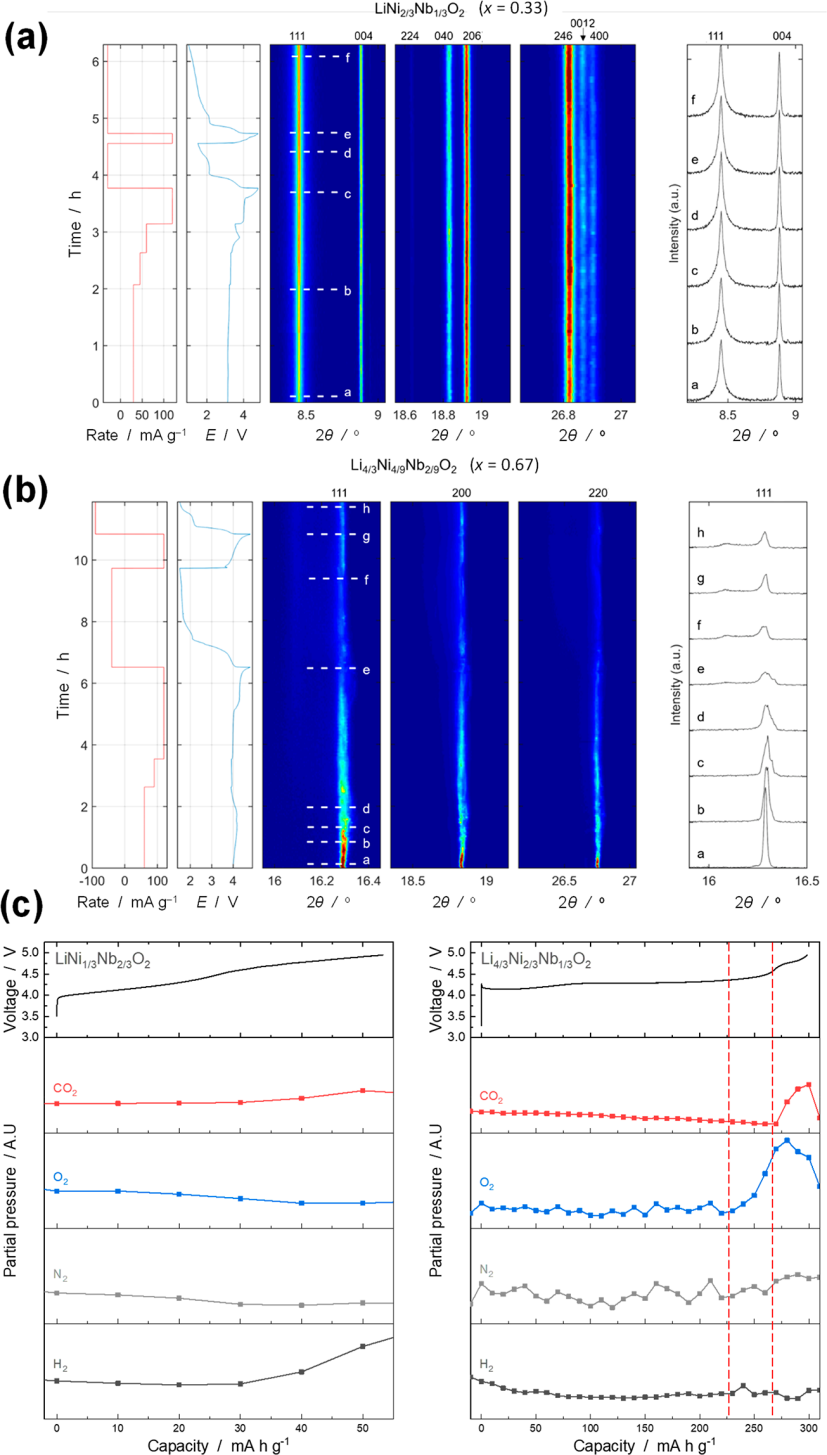
Selected regions of the *in operando* synchrotron XRD contour maps at 50 °C of the first two complete electrochemical cycles for (a) LiNi_2/3_Nb_1/3_O_2_ (*x* = 0.33) and (b) Li_4/3_Ni_2/9_Nb_4/9_O_2_ (*x* = 0.67). The corresponding electrochemical load curves (current rate and voltage) are shown on the left. A new rocksalt phase with a low crystallinity appeared upon charge for the Li-excess sample. Also see the Supporting Information, including Supporting Figures S9 and S10, for more details. The same samples shown in Figure 2 were used for the analysis. (c) *In situ* partial gas pressures of CO_2_, H_2_, N_2_, and O_2_ from LiNi_2/3_Nb_1/3_O_2_ and Li_4/3_Ni_2/9_Nb_4/9_O_2_ and the corresponding charge curves obtained at room temperature.

To clarify differences found in the electrochemical properties, charge compensation and phase evolution processes of two structurally and compositionally distinct rocksalt phases, namely stoichiometric LiNi_2/3_Nb_1/3_O_2_ (*x* = 0.33) and Li-excess Li_4/3_Ni_2/9_Nb_4/9_O_2_ (*x* = 0.67), were examined by *ex situ* and *in operando* XRD, OEMS, and *ex situ* XAS, as shown in Figures 1 and 2, respectively. The results of the *ex situ* XRD analysis are summarized in Supporting Section S1 and Supporting Figure S8. Highly reversible changes in the crystal structure of stoichiometric Li_1–*y*_Ni_2/3_Nb_1/3_O_2_ were noted from the XRD data during the charge–discharge process, whereas complicated phase evolution was observed for Li-excess Li_4/3–*y*_Ni_2/9_Nb_4/9_O_2_. These trends are further supported by the *in operando* synchrotron XRD experiments conducted for both oxides at 50 °C (see the Supporting Information). A contour map of Li_1–*y*_Ni_2/3_Nb_1/3_O_2_ (*x* = 0.33) obtained using *in operando* XRD is shown in Supporting Figure S9a, and enhancements of selected 2θ regions are plotted in Figure 3a. Similarly, a contour map and analyzed data of Li_4/3–*y*_Ni_2/9_Nb_4/9_O_2_ (*x* = 0.67) are also shown in Figure 3a and Supporting Figure S10. The samples exhibit quite different behaviors during electrochemical cycling. The *in operando* XRD study clearly reveals that LiNi_2/3_Nb_1/3_O_2_ shows good structural stability, while the Li-rich Li_4/3_Ni_2/9_Nb_4/9_O_2_ undergoes irreversible structural degradation. The irreversible phase transition of Li_4/3–*y*_Ni_2/9_Nb_4/9_O_2_ is accompanied by the formation of a range of secondary isostructural phases, which are likely (partially) delithiated phases with different lattice parameters, crystallinities, and local strains. Closer inspection of the *in operando* XRD data (Figure 3a and Supporting Figure S10c and d) reveals that this involves a series of complex mechanisms or phase transitions, possibly including partial amorphization.

Gas generation related to phase evolution was measured by online electrochemical mass spectrometry (OEMS),^50^ as shown in Figure 3c. Because excessive electrolyte vaporization at elevated temperatures resulted in the difficulty during the OEMS study, the measurement was conducted at room temperature. A smaller charge capacity with a larger polarization was observed for stoichiometric LiNi_2/3_Nb_1/3_O_2_ (*x* = 0.33) at room temperature. An increase in the partial pressure of hydrogen, which originates from electrolyte decomposition, was observed at the higher voltage region (>4.5 V), but the evolution of O_2_ and CO_2_ gas was not detected. The diffusion of protic species generated by electrolyte oxidation from the positive electrode side to the metallic lithium negative electrode side and their subsequent reduction by metallic lithium result in hydrogen generation.^51^ In contrast, oxygen generation was clearly observed for Li_4/3–*y*_Ni_2/9_Nb_4/9_O_2_ (*x* = 0.67) upon charge. An increase and decrease in the partial pressures of CO_2_ and O_2_, respectively, were observed at the higher voltage region, suggesting that oxygen released from the oxide upon charge further reacts with the electrolyte. Such irreversible reactions, including oxygen loss, are expected to be enhanced at elevated temperatures. Although the gas species generated in Li cells was qualitatively analyzed in this study, future work will target the use of a more quantitative approach^29^ to OEMS.

**Figure 4:**
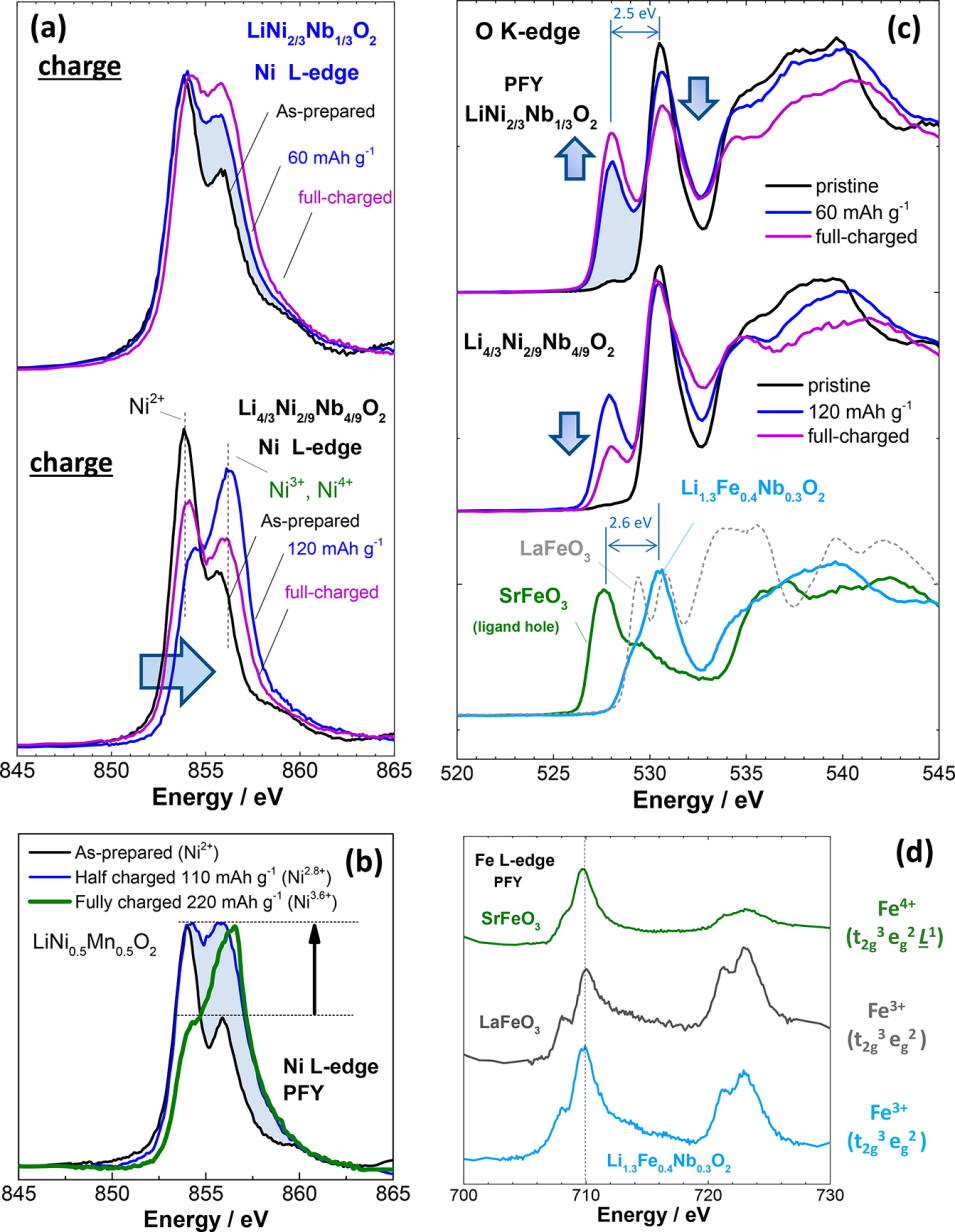
Variations of electronic structures of Ni and O in the electrochemical charge–discharge processes. (a) Change in the Ni L edge spectra of Li_1–*y*_Ni_2/3_Nb_1/3_O_2_ (*x* = 0.33) and Li_4/3–*y*_Ni_2/9_Nb_4/9_O_2_ (*x* = 0.67). (b) Change in the Ni L edge spectra of Li_1–*y*_N*i*_1/2_M*n*_1/2_O_2_. (c) Change in the O K edge XAS spectra of Li_1–*y*_Ni_2/3_Nb_1/3_O_2_ and Li_4/3–*y*_Ni_2/9_Nb_4/9_O_2_. As shown in blue shaded areas in panels a and c, for Li_1–*y*_Ni_2/3_Nb_1/3_O_2_, a more pronounced change was noted for the O K edge spectra compared with the Ni L edge spectra upon the initial charge to 60 mA h g^–1^. O K edge XAS spectra of SrFeO_3_ and LaFeO_3_, which were used as reference materials for anionic redox, are also shown in panel c. The same samples shown in Figure 2 were used for the analysis. Changes in the O K edge XAS spectra for layered LiNi_1/2_Mn_1/2_O_2_ as a reference material are shown in Supporting Figure S12, and the results for disordered Li_1–*y*_Ni_2/3_Nb_1/3_O_2_ and layered Li_1–*y*_Ni_1/2_Mn_1/2_O_2_ are compared in Supporting Figure S13. (d) Comparison of Fe L edge spectra of SrFeO_3_, LaFeO_3_, and Li_1.2_Fe_0.4_Nb_0.3_O_2_^55^ with that of a disordered rocksalt structure.

Similar trends were also noted for the Ni K edge XAS data (Supporting Figure S11). For both compositions, the spectra of the as-prepared samples were found at an energy similar to that of divalent Ni ions. For Li_1–*y*_Ni_2/3_Nb_1/3_O_2_, the Ni K edge XAS spectra slightly shift to a higher energy region as charge capacity increases, indicating the partial oxidation of Ni^2+^. Nevertheless, the shift of spectra is unexpectedly small when compared with that of a reference material of Ni^3+^ (LiNiO_2_). This trend is also supported from the observation of Ni L edge spectra with the fluorescence yield mode (Figure 4a). Some extent of Ni oxidation was observed even after Li_1–*y*_Ni_2/3_Nb_1/3_O_2_ was charged to 4.8 V, but changes in the spectra are small compared with those of layered Li_1–*y*_Ni_1/2_Mn_1/2_O_2_ (Figure 4b). A much smaller change in the area of the XAS spectra was observed when compared with tat of the reference material, *i.e*., half-charged LiNi_1/2_Mn_1/2_O_2_ with Ni^2.8+^. These observations suggest that the Ni ions remain at a lower average oxidation state (at least less than 3+) even after being charged to 4.8 V (also see Supporting Figures S12 and S13). This trend is further supported by the analysis of the extended X-ray absorption fine structure (EXAFS) spectra, as shown in Supporting Figure S14. The formation of Ni^3+^ ions, which are Jahn–Teller active,^52^ is less evident for the stoichiometric sample even after charging. Moreover, after discharge to 1.5 V, the energy and profile of the spectrum are identical to that of the as-prepared sample. In addition, no change in the Nb K edge XAS spectra was noted during charging and discharging (Supporting Figure S15). The high reversibility of the XAS spectra at the Ni K edge is consistent with the XRD data and clearly shows that the contribution from Ni and Nb ions is unexpectedly small. As a result, the cationic redox reaction of Ni ions alone cannot explain the reversible redox reaction observed in Figure 2.

In contrast, complicated changes were observed for Li-excess Li_4/3–*y*_Ni_2/9_Nb_1/3_O_2_. When the sample was charged to ∼120 mA h g^–1^, the Ni K edge and L edge XAS spectra shifted to higher-energy regions, indicating the oxidation of the Ni ions. The average oxidation state is clearly higher than that of the Ni ions in the fully charged stoichiometric sample. When the sample was further charged to 4.8 V, the reduction of Ni ions was clearly evidenced in the Ni L edge XAS spectra. This is similar to the reduction of Ni upon “charge” that is associated with oxygen loss, which was reported in Li_2_MnO_3_-based positive electrodes.^53,54^ Moreover, for the Li-excess system, the XAS spectrum at the Ni K edge shifts back to the lower energy region after discharge to 120 mA h g^–1^ and is found at nearly the same energy as the as-prepared sample. Upon further discharge, a drastic change is found in the spectrum, whose energy is lower than that of the as-prepared sample.When the spectrum of a Ni foil was compared for reference, it was thought that Ni^2+^ was partially reduced to a metallic state. From the Ni K edge spectra, it was expected that approximately 30% of Ni ions were reduced to the metallic state. This consideration is further supported by analysis of the EXAFS spectra (Supporting Figure S14). Similar to Li_4/3_Ni_2/9_Nb_1/3_O_2_, the reduction of Fe after discharge has been reported in the Fe system because of unavoidable oxygen loss upon charge.^54,55^

Nanostructures of the particles after electrochemical cycling were observed by TEM experiments. For stoichiometric Li_1–*y*_Ni_2/3_Nb_1/3_O_2_, no change was observed after electrochemical cycling, and high crystallinity was noted from its electron diffraction pattern (Supporting Figure S16a inset) after the sample was discharged to 1.5 V. In contrast, for Li-excess Li_4/3–*y*_Ni_2/9_Nb_1/3_O_2_, significant differences were observed before and after electrochemical cycling (Supporting Figure S16b). In the cores of the particles, a high crystallinity region with clear lattice fringes such as observed in the as-prepared sample was still found. However, the lattice fringes disappear at the edge of particles after discharge. A reduction of crystallinity at the edge and the formation of nanosized particles (less than 5 nm) were observed in the SAED pattern. These findings clearly indicate that a conversion reaction (NiO + 2Li^+^ + 2e^–^ → Ni + Li_2_O) proceeds upon discharge, as expected from the XRD/XAS data, and also suggest that oxygen release occurs upon charge. The formation of metallic Ni is consistent with the results from the Ni K edge spectra after discharge. The reduction of Ni ions and the formation of Li_2_O are also supported by the analysis of the Ni L edge and O K edge XAS spectra (Supporting Figure S17). Note that the reduction of Nb upon discharge is not evidenced by the XAS spectra (Supporting Figure S15). Following the charge, a new voltage plateau was observed at around 2 V (Figure 2a);a similar voltage profile was observed for nanosized NiO in Li cells with a conversion reaction.^56^ Reoxidation of metallic or nanosized Ni results in the appearance of this new plateau at 2 V, coupled with larger polarization upon further charge, and thus poor reversibility as an electrode material, as shown in Figure 2d. Similar to this work, the formation of amorphous phases near the surfaces of the particles was reported in the Li_2_MnO_3_ system and found to be associated with irreversible oxygen loss during electrochemical cycling.^57^

### Unexpectedly Large Contribution of Oxygen to Charge Compensation during Electrochemical Oxidation

For the stoichiometric phase, the contribution of Ni to charge compensation was unexpectedly limited. O K edge XAS spectra of these samples were, therefore, collected and analyzed. For stoichiometric layered materials containing Ni, for instance, LiNiO_2_^58^ and LiCo_1/3_Ni_1/3_Mn_1/3_O_2_,^59^ some extent of peak shifting was observed in the O K edge XAS spectra after Li extraction, but minor changes were generally observed. O K edge XAS spectra of layered Li_1–*y*_Ni_1/2_Mn_1/2_O_2_ measured at the same beamline are also shown in Supporting Figure S12. The shift in energy is correlated with effective nuclear charges associated with the electrochemical oxidation of Ni, which hybridizes with the O 2p orbital to form σ-like bonds. In contrast to this observation, more pronounced changes in the profiles of the XAS spectra were observed in this binary system, as shown in Figure 4c. A new peak centered at 528 eV appears in the O K edge XAS spectra of stoichiometric Li_1–*y*_Ni_2/3_Nb_1/3_O_2_, and this peak grows as the charge capacity increases. A large difference in energy (2.5 eV) was observed for the new peak compared with the original peak centered at 530.5 eV. Note that this peak is clearly visible in the fluorescence yield mode, which is bulk-sensitive (see Supporting Figure S18). The change in the oxidation state of the Ni ions for the stoichiometric sample was small after the sample was charged to 60 mA h g^–1^, as shown in Figure 4a, whereas a clear peak at 528 eV was observed in the O K edge XAS spectrum, as shown in Figure 4c. The appearance of this peak suggests that additional unoccupied states were formed in the O 2p orbital by electrochemical oxidation, *i.e*., anionic redox and charge compensation by oxygen. It is noted that this peak at 528 eV was also observed for the Li-excess system after the sample was charged to 120 mA h g^–1^, but the intensity of this peak decreased after the same was further charged to 4.8 V. This is consistent with the reduction of Ni ions upon charge (see Figure 4a and the K edge XAS spectra shown in Supporting Figure S11) and is most likely associated with oxygen loss for the Li-excess system, indicating that anionic redox is energetically destabilized for the Li-excess system. On the basis of these observations, it is proposed that a larger contribution to the charge compensation by anionic species was achieved, especially for the stoichiometric sample.

### Experimental and Theoretical Analysis of Oxygen-Unoccupied States with Model Materials

In this study, SrFeO_3_ with nominal Fe^4+^ prepared by the high-pressure method was selected as a model material with an unoccupied state of oxygen. The term “ligand hole” (t_2g_^3^e_g_^1^ ≈ t_2g_^3^e_g_^2^*L*^1^) is also used to describe the unique electronic structure of oxygen in perovskite-type SrFeO_3_.^60^ In general, the oxygen0unoccupied state is not energetically stable, but the employed high-pressure approach allows us to synthesize SrFeO_3_. For comparison, LaFeO_3_ was also used as reference for Fe^3+^, as it possesses the same perovskite structure as SrFeO_3_. XRD patterns of as-prepared SrFeO_3_ and LaFeO_3_ are shown in Supporting Figure S19. The refined structural parameters are found in Supporting Table S3 and S4. The comparison of the XAS spectra of SrFeO_3_ and LaFeO_3_ clearly reveals that both perovskite oxides have quite similar profiles in the Fe L edge XAS spectr (Figure 4d), indicating that both Fe ions have an electronic structure similar to the high-spin d^5^ state (t_2g_^3^e_g_^2^). In contrast, a significant difference was noted for the O K edge XAS spectra in Figure 4c. The energy of the pre-edge peak for SrFeO_3_ is much lower (∼2.6 eV) when compared with that of LaFeO_3_. Moreover, the pre-edge peak observed for SrFeO_3_ is close to the new peak found in Li_1–*y*_Ni_2/3_Nb_1/3_O_2_ after electrochemical oxidation. A similar trend in the O K edge spectra was also observed for La_1–*x*_Sr_*x*_CrO_3_, where hole formation due to Sr substitution resulted in the transition from an insulator to a metal.^61^

**Figure 5:**
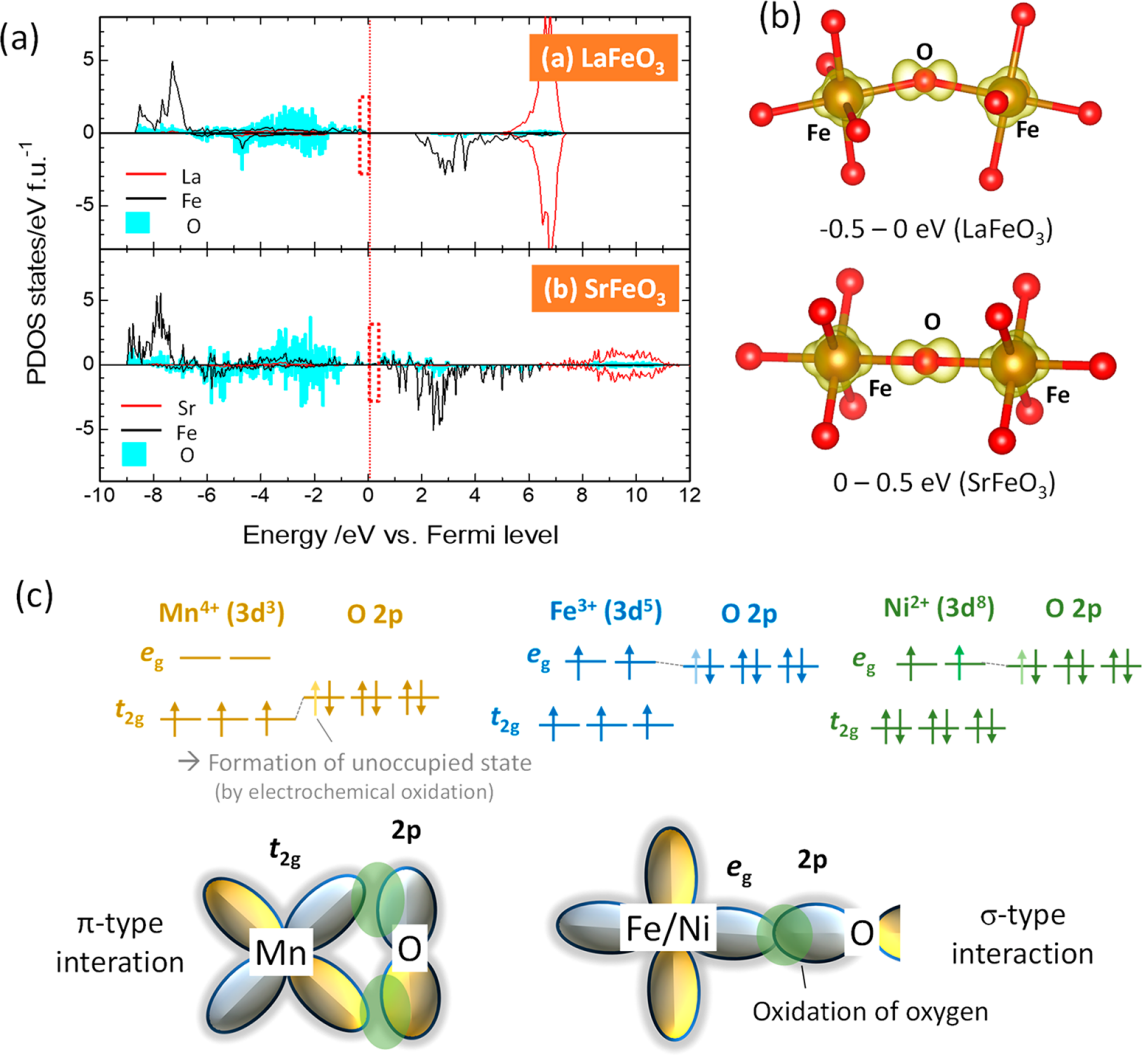
(a) Partial density of states (PDOS) of (a) LaFeO_3_ and SrFeO_3_. Positive and negative values for the PDOS indicate majority and minority spin states, respectively. (b) Visualized partial density of electronic states for the Fe–O–Fe arrangement in LaFeO_3_ and SrFeO_3_ near the Fermi level. The energy ranges from −0.5 to 0 vs the Fermi level and from 0 to 0.5 eV vs Fermi level for LaFeO_3_ and SrFeO_3_, respectively. The corresponding energy regions are indicated by red dotted lines in the panel a. (c) Based on the experimental observation of the O K edge spectrum of SrFeO_3_ (Figure 4c), the pre edge peak at 527.5 eV corresponds to the formation of the unoccupied state of oxygen, which is stabilized through σ-type interactions with e_g_ electrons of Fe ions as visualized in panel b. Electron configurations of Mn^4+^, Fe^3+^, and Ni^2+^ in the *O*_*h*_ symmetry and schematic illustrations of π- and σ-type bonds are also shown.

To further study the electronic structures of LaFeO_3_ and SrFeO_3_, electron distributions of LaFeO_3_ and SrFeO_3_ were calculated using the Heyd–Scuseria–Ernzerhof (HSE06) hybrid functional calculation, and the results are summarized in Figure 5. Calculated Bader charges and net spin moments^62^ of LaFeO_3_ and SrFeO_3_ are also summarized in Table 1. Similar Bader charges were observed for the Fe ions of both oxides, while a lower negative charge for oxygen is observed in SrFeO_3_. The net spin moment of Fe also decreased from 4.15 for LaFeO_3_ to 3.71 for SrFeO_3_. In addition, from the PDOS (Figure 5a) above the Fermi level (0–0.5 eV) and its calculated electron density (Figure 5b), relatively larger contributions of both Fe and O were noted for SrFeO_3_. In the case of Mn^4+^–O bonds in disordered rocksalt-type Li_1.3-x_Nb_0.3_Mn_0.4_O_2_, the PDOS just above the Fermi level mainly consists of O ions, with a minor contribution of manganese ions.^55^ Such character originates from hole stabilization by a relatively weak π-type interaction between the Mn t_2g_ and O 2p orbitals (see Figure 5c).^44^ In contrast, a strong σ-type interaction between Fe and O was observed for SrFeO_3_, as shown in Figure 5b, indicating that the oxygen-unoccupied state is energetically stabilized by the formation of a chemical bond between the Fe e_g_ and O 2p orbitals. If a similar electron configuration occurs in the Ni system, the similar σ-type interaction with oxygen is anticipated.

**Table 1:** Calculated Bader Charges and Net Spin Moments of LaFeO_3_ and SrFeO_3_^a^

	Bader charge		net spin moment (μ_B_)	
atom	LaFeO_3_	SrFeO_3_	LaFeO_3_	SrFeO_3_
La or Sr	2.18	1.61	0.00	0.00
Fe	1.92	1.91	4.26	3.71
O	–1.37	–1.17	0.25	0.11

a La, Sr, and O (in LaFeO_3_) ions are La^3+^, Sr^2+^, and O^2–^, respectively, due to net-zero spin moments. Hole creation associated with the replacement of the La^3+^ ion (LaFeO_3_) by the Sr^2+^ ion (SrFeO_3_) occurs at both oxide ions and Fe ions due to change of Bader charges and net spin moments.

### Theoretical Understanding of Anionic Redox in Li_1–*y*_Ni_2/3_Nb_1/3_O_2_

#### The Structural Model of Li_1–y_Ni_2/3_Nb_1/3_O_2_ Used for Computation

The genetic algorithm (GA) approach^63^ was used to explore energetically favorable cation arrangements for LiNb_1/3_Ni_2/3_O_2_ and the delithiated phases Li_1–*x*_Nb_1/3_Ni_2/3_O_2_ (*x* = 0.17, 0.33, 0.50, 0.67, and 1.00). Details of the GA approach are described in Supporting Section S2. The superstructure model Li_18(1–*x*)_Nb_6_Ni_12_O_36_ with a rocksalt structure is considered in the present GA procedure. The lattice vectors *a*_sc_, *b*_sc_, and *c*_sc_ in the supercells correspond to linear combinations of lattice vectors *a*_p_, *b*_p_, and *c*_p_ in a primitive cell with a rocksalt structure, where (*a*_sc_*b*_sc_*c*_sc_) = (*a*_p_*b*_p_*c*_p_) *M*_sc_. The supercell matrix *M*_sc_ was set as ash shown below to achieve agreement between experimental and computational compositions with reasonable computational costs.
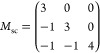


It was also confirmed that the calculated Li removal voltage for this supercell was 0.08 V higher than that for the cell four-times larger than the original cell. Note that total energies of the models were evaluated during the GA procedure using DFT calculations with the GGA + *U* functional. As the calculation of total energies for the different models, around several thousand configurations, was needed, the DFT approach was the most economical. The *U* value for the Ni 3d states was set as 6.0 eV based on previous papers.^64,65^ After GA cycles, the five lowest-energy configurations obtained from GA at each composition were recalculated using the HSE06 functional to estimate formation energies of delithiated systems.

**Figure 6:**
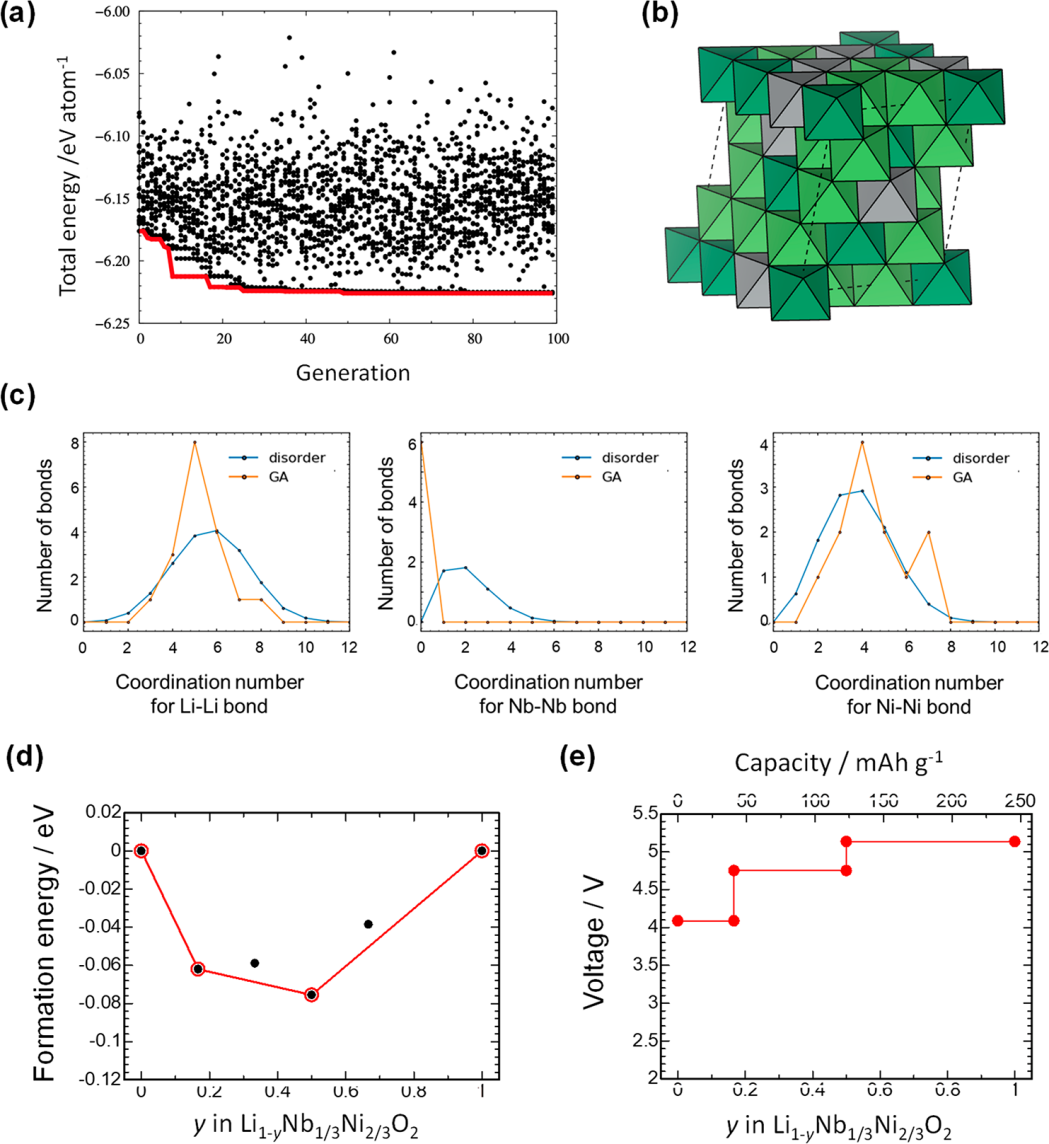
Structural optimization of LiNi_2/3_Nb_1/3_O_2_ for theoretical calculations. (a) Total energy variations of Li_18(1–*y*)_Ni_12_Nb_6_O_36_ as a function of generation in the genetic algorithm (GA) procedure (also see Supporting Section S2 for the GA procedure). The red line indicates the lowest energy in each generation. (b) A schematic illustration of the crystal structure of Li_18_Ni_12_Nb_6_O_36_ obtained from GA, with partial cation ordering for Nb and disordered Li/Ni arrangements. Light green, dark green, and gray colored octahedra denote LiO_6_, NbO_6_, and NiO_6_, respectively. Dotted lines correspond to a unit cell of Li_18_Ni_12_Nb_6_O_36_. (c) Distributions of the coordination number for NN Li–Li, Nb–Nb, and Ni–Ni bonds for LiNi_2/3_Nb_1/3_O_2_, which were derived from GA optimization and cation-disordered models. (d) Formation energies of Li_1–*y*_Nb_1/3_Ni_2/3_O_2_ estimated from first-principles calculations with the HSE06 hybrid functional. The formation energies are normalized by each formula unit. (e) Calculated voltage profiles of Li_1–*y*_Ni_2/3_Nb_1/3_O_2_.

Figure 6 shows (a) the energy distribution during the GA optimization and (b) the optimized structure of LiNi_2/3_Nb_1/3_O_2_. Crystallographic information on LiNi_2/3_Nb_1/3_O_2_, which was derived using the GA optimization, is also shown in Supporting Table S5. Figure 6c also shows distributions of the coordination number of nearest-neighboring (NN) Li–Li, Nb–Nb, and Ni–Ni bonds in LiNi_2/3_Nb_1/3_O_2_, which were obtained using the GA-optimized structure (orange lines). Corresponding distribution profiles originating from the disordered structure, modeled using 36 000 cations sites, are presented (blue lines) for comparison. In the GA-derived structure, there is no NN Nb–Nb bond formation, whereas the disordered structure shows almost no isolated Nb ions. This fact indicates that Nb ions are structurally ordered in the GA-optimized structure. On the other hand, coordination number distributions for Li–Li and Ni–Ni interactions in the GA-derived structure are similar to those in the disordered structures. These structural features agree with the experimentally observed structure, where partial Nb ordering was observed for the sample prepared with a short heating time (Supporting Figure S6) while Ni and Li ions were essentially disordered, as mentioned previously. Thus, it was concluded that the GA-derived structure model was more appropriate in this study than a fully disordered structure.

Total energy changes of delihiated phases of Li_1–*y*_Nb_1/3_Ni_2/3_O_2_ were further computed. It is noted that only the Li/vacancy arrangement was considered in GA optimization of the delithiated phases because the migration of multivalent cations, namely Ni and Nb ions, is unlikely during electrochemical delithiation at 50 °C. The relaxed structures with the GA-optimized Li/vacancy arrangement shown no dimerization of oxygen atoms over the entire range of composition *y* (0 ≤ *y* ≤ 1 in Li_1–*y*_Nb_1/3_Ni_2/3_O_2_), as the minimum bond distance between two oxygen atoms is 2.53 Å (Supporting Figure S20). The phase stability of partially delithiated samples was evaluated by plotting the formation energies, *ΔE*_f_, using energies of fully lithiated and delithiated compositions as references.^76,66^
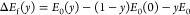
1where *E*_0_(*y*) corresponds to total energy of the compound with composition *y* evaluated using first-principles calculations with the HSE06 functional. Formation energies of the corresponding structures obtained using eq 1 are plotted in Figure 6d. Circle symbols indicate structures that are the most energetically stable at composition *y*. Tie lines in the figure represent the convex hull among the GA-optimized structures. The figure shows that several intermittent compositions of the calculated Li_1–*y*_Nb_1/3_Ni_2/3_O_2_ structures are thermodynamically stable, indicating that solid solution phases are formed upon delithiation; this is also consistent with the experimental finding. Delithiation voltages were also estimated according to the following methodology used in the literature:^67^
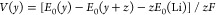
2where *F* stands for the Faraday constant and *E*_0_(*y* + *z*) and *E*_0_(Li) correspond to total energies of the compound with composition *y* + *z* and the Li metal, respectively. It is noted that the voltages resulting from the computation (Figure 6e) range from ∼4 to ∼5 V, also showing agreement with the experimental results.

#### Analysis of Charge Compensation Mechanisms from Changes in the Partial Density of States

**Figure 7:**
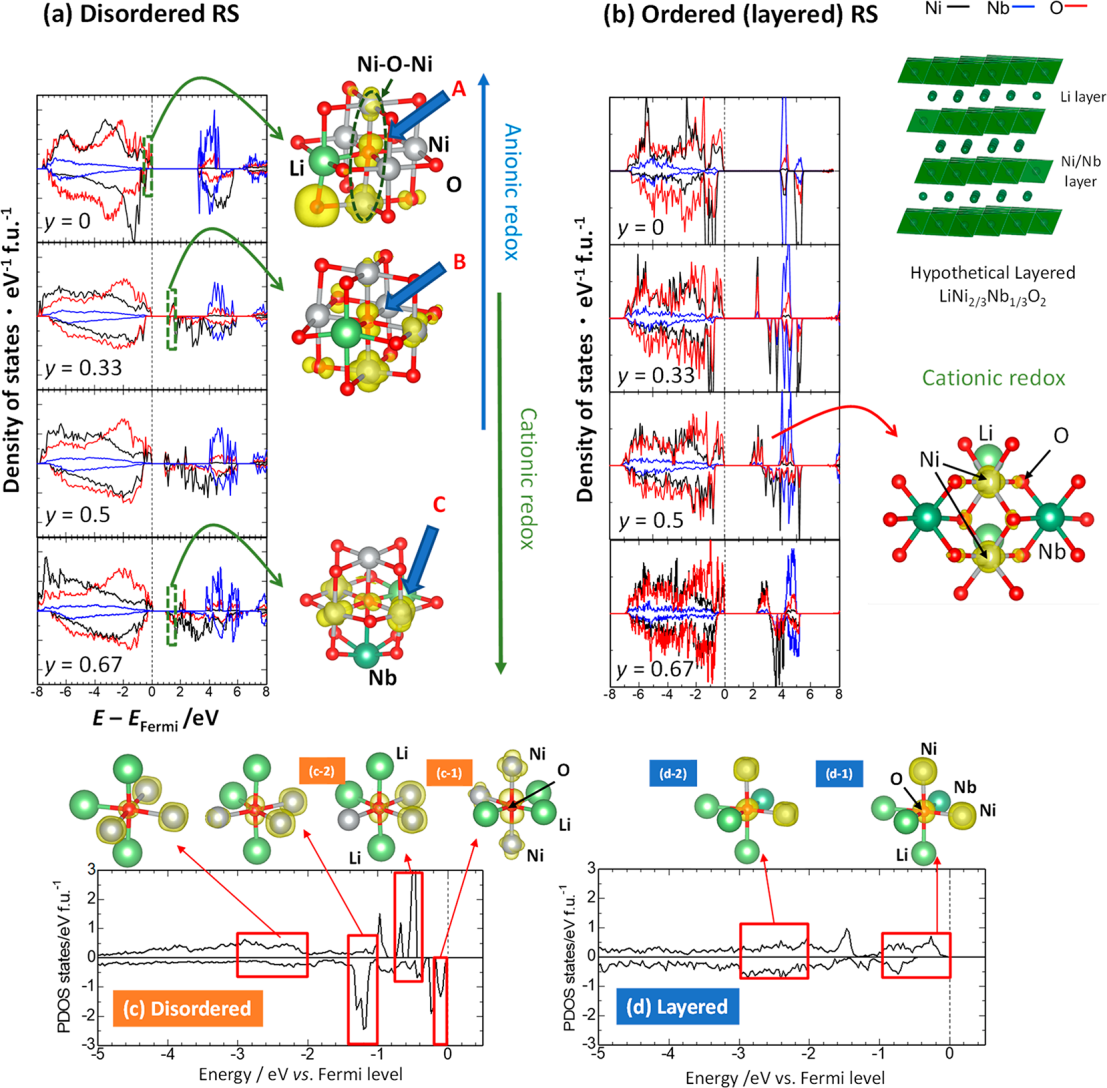
Electronic structure study of LiNi_2/3_Nb_1/3_O_2_ with the cation disordered structure using DFT calculations. (a) PDOS results of disordered Li_1–*y*_Ni_2/3_Nb_1/3_O_2_ at different states of charge. Black, blue, and red lines indicate the PDOS for Ni, Nb and O ions, respectively. The visualized partial density of states for different compositions is also shown. (b) Data for hypothetical layered Li_1–*y*_Ni_2/3_Nb_1/3_O_2_. PDOSs for specific oxide ions in (c) disordered and (d) hypothetical layered LiNi_2/3_Nb_1/3_O_2_ along with visualized electronic configurations. See the text for more details.

The partial density of states (PDOS) of each orbital and the partial electronic state densities for occupied and unoccupied states at several PDOS peaks for Li_1–*y*_Ni_2/3_Nb_1/3_O_2_ are shown in Figure 7a. For the stoichiometric phase *y* = 0, the PDOS near the Fermi level consists of both of O 2p and Ni e_g_ hybridized orbitals (arrow A in Figure 7a). Moreover, a linear Ni–O–Ni configuration with an antibonding character was found. This unique configuration was realized only for the disordered structure and was not formed for the layered structure. After Li extraction, *y* = 0.33, the PDOS of Ni 3d hybridized with O 2p orbitals appears at the bottom of the unoccupied states (arrow B in Figure 7a). Corresponding partial electron and hole densities clearly indicate the σ-type hybridization of the Ni 3d and O 2p orbitals. Even at the initial stage of charge, an obvious change was observed in the O 2p PDOS, which is consistent with the experimental observation. A contribution to the Ni 3d orbital from both sides of the O 2p orbital was noted, but the pronounced presence of the O 2p orbital was also observed, indicating the formation of an unoccupied state for oxygen with the partial oxidation of Ni ions. This electronic configuration is expected to be analogous to that in SrFeO_3_ (Figure 5) and is a σ-type interaction between Ni and O. Note that a linear Fe–O–Fe configuration is also formed in perovskite-type SrFeO_3_. Further delithiation resulted in a different oxygen contribution trend, and an increase in the relatively large contribution from Ni ions was found in the PDOS for *y* = 0.67 (arrow C in Figure 7a). Cationic redox is more pronounced in the later delithiation process, which is further discussed in the next section.

To further emphasize the uniqueness of the linear Ni–O–Ni configuration formed in the disordered structure, electronic structures of a hypothetical material, “layered” LiNi_2/3_Nb_1/3_O_2_, were computed, and the PDOSs of the layered and disordered samples are compared in Figure 7b (also see Supporting Section S3 and Supporting Figure S21). Figure 7b shows variations of the PDOSs of Ni, Nb, and O ions with respect to the composition *y*. The top of the valence bands mainly consists of Ni 3d and O 2p orbitals for the entire compositional range, indicating the hybridization of the Ni–O bonds. For comparison purposes, PDOSs of a specific oxide ion near the Fermi level were also computed for both disordered and ordered (layered) LiNi_2/3_Nb_1/3_O_2_, and the electron densities are visualized in Figure 7c and d, respectively. For layered LiNi_2/3_Nb_1/3_O_2,_ the linear Ni–O–Ni configuration was not formed, and a clear bonding formation between Ni and O was not found (marked as “d-1” in Figure 7d). In contrast, for disordered LiNi_2/3_Nb_1/3_O_2_, the PDOS near the Fermi level (−0.5–0 eV) mainly consists of ions with the linear Ni–O–Ni configuration and a σ-type bond (marked as “c-1” in Figure 7c); thus, the contribution of oxygen is more pronounced compared with that in layered LiNi_2/3_Nb_1/3_O_2_. Moreover, a nonlinear Ni–O–Ni configuration (marked as “c-1”) and a linear Li–O–Li configuration (marked as “c-2”) were also found in disordered LiNi_2/3_Nb_1/3_O_2_, but the energy relative to the Fermi level was lower for the linear Ni–O–Ni configuration. The linear Li–O–Li configuration with a nonbonding character is energetically stable compared with the linear Ni–O–Ni configuration with an antibonding character. After the delithiation of the layered phase, components consisting of Ni and O orbitals are visible at the bottom of the conduction bands. This was also confirmed by the visualized images of DOS in Figure 7b, where the top of the valence band is at *y* = 0.5 and the bottom of the conduction band (unoccupied states) is at *y* = 0.67. In both images, larger contributions from Ni 3d orbitals are visible, and the contributions from the surrounding oxide ions are reduced when compared with those in the disordered phase in the early stage of charge.

**Figure 8:**
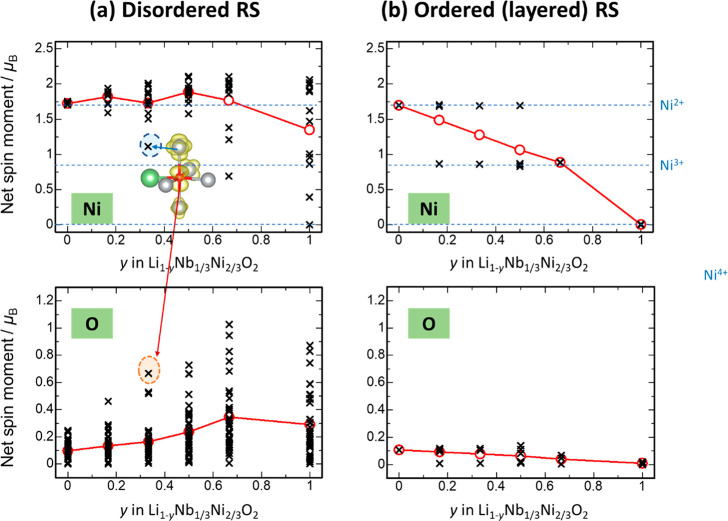
Estimated changes in the oxidation states of Ni and O ions during Li extraction from net spin moments. The data for (a) disordered LiNi_2/3_Nb_1/3_O_2_ and (b) hypothetical layered LiNi_2/3_Nb_1/3_O_2_ are shown. The net spin moments are defined as the difference between the up and down electron spins as a function of the integration radius (1.2 Å in this study) of the corresponding ion cores. Changes in the net spin moments for LiNiO_2_ and NiO_2_ are also shown in Supporting Figure S23. Red lines in the figure correspond on average to changes in the net spin moments.

Similar to SrFeO_3_, net spin moments for Ni and O ions upon delithiation were computed to further estimate charge compensation mechanisms, and the results for the disordered and hypothetical layered samples are summarized in Figure 8. Figure 8a shows changes in the net spin moments of the Ni and O ions in disordered RS-type Li_1–*y*_Ni_2/3_Nb_1/3_O_2_ (*y* = 0, 0.17, 0.33, and 0.50). The majority of the net spin moments of Ni ions at composition *y* = 0 are distributed around >1.5, corresponding to the formal oxidation state of Ni, *i.e*., 2+ (t_2g_^6^ e_g_^2^), and the net spin moments of several Ni ions decrease to ∼1.2 after the delithiation. Note that the net spin moments of some of Ni ions are reduced to *ca*. 1.2 for *y* = 0.17–0.33. These Ni ions originate from the partial oxidation of Ni ions in the linear Ni–O–Ni configuration. Nevertheless, the net spin moments of Ni ions are on average higher than those found for Ni^3+^ in the layered structure, as shown below. Moreover, the Ni oxidation states for *y* = 0.33 are on average (shown as a red line in the figure) close to those for *y* = 0. Relatively larger changes in Ni oxidation states from the spin moments are expected for *y* > 0.33, owing to the oxidation of Ni ions in nonlinear configurations. Complicated changes in the net spin moments indicate that many different local configurations of Ni ions are formed in the disordered structure, as shown in Figure 7c.

On the other hand, net spin moments of O ions are distributed around zero for stoichiometric and disordered RS-type LiNi_2/3_Nb_1/3_O_2_, corresponding to a closed-shell electronic configuration, *i.e*., oxide ions, O^2–^. The electrochemical Li extraction causes a gradual increase in the net spin moments of the O ions, as shown in Figure 8, indicating both the oxidation of the O^2–^ ions and the lack of oxygen dimerization^68^ for relaxed structures, as mentioned before. Note that oxide ions with relatively larger spin moments are found in oxygen with a linear Ni–O–Ni configuration (see the red hatched circle in Figure 8a). Moreover, the changes in the net spin moments for oxygen are unique for Li_1–*y*_Ni_2/3_Nb_1/3_O_2_ with the cation-disordered structure, and a similar observation was noted for SrFeO_3_ with the linear Fe–O–Fe configuration (Figure 5b).

In contrast to the disordered structure, hypothetical layered Li_1–*y*_Ni_2/3_Nb_1/3_O_2_ has net spin moments for Ni ions that are discretely distributed and reduced from 1.6 to 0.8 at the compositional range of 0 ≤ *y* ≤ 0.67, which corresponds to oxidation of Ni^2+^ (t_2g_^6^ e_g_^2^) to Ni^3+^ (t_2g_^6^ e_g_^1^) because net spin moments for N^i2+^ and Ni^3+^ are expected to be 2 and 1, respectively. Further delithiation at *y* > 0.67 causes the oxidation of Ni^3+^ to Ni^4+^ while the net spin moments for oxide ions are almost zero for the entire compositional range. Some of the oxide ions have nonzero spin moments (<0.1), owing to the strong hybridization between Ni and O ions. The calculated high-voltage profile (>5 V) at *y* > 0.67 in Supporting Figure S21b is ascribed to Ni^3+/4+^ oxidation, and the contribution of O^2–^ oxidation is excluded on the basis of the net spin moments, which are clearly different from those of the cation-disordered rocksalt sample with the linear Ni–O–Ni configuration. Moreover, the average net spin moments of Ni ions are much higher for the the disordered structure, indicating the Ni oxidation states are lower. Supporting Figure S22 shows variations of Barder charges for Ni and O ions in (a) disordered and (b) ordered (layered) RS-type Li_1–*y*_Ni_2/3_Nb_1/3_O_2_. The trend of Bader charges supports the above results of the net spin moment shown in Figure 8. For example, the oxidation of several oxide ions is clearly visible in disordered Li_1–*y*_Ni_2/3_Nb_1/3_O_2_, while no significant variation of Bader charges for oxide ions is indicated at the same composition *y* in ordered Li_1–*y*_Ni_2/3_Nb_1/3_O_2_. This indicates the importance of the local coordination environment for each oxide ion, though averaged Bader charges for both Ni and O gradually change due to the strong hybridization between the Ni 3d and O 2p orbitals.

Such uniqueness of the linear Ni–O–Ni configuration for the disordered phase is further evidenced by the theoretical calculation of layered NiO_2_, which was obtained via the oxidation of layered LiNiO_2_ without Nb ions (Supporting Figure S23). After delithiation from layered LiNiO_2_, zero-spin moments of O ions were found in NiO_2_. This fact clearly indicates that the presence of Nb ions is not responsible for the large spin moments of the O ions. The zero-spin moments of oxygen were found for both layered NiO_2_ and Ni_2/3_Nb_1/3_O_2_ without the linear Ni–O–Ni environment. On the basis of these results, it was proposed that the formation of the linear Ni–O–Ni configuration is triggered by structural disordering, where the presence of Nb ions is essential to activate the anionic redox for Ni-based oxides. Note that approximately 10% anti-site defects form in LiNi_1/2_Mn_1/2_O_2_,^27^ leading to the formation of the liner Ni–O–Ni environment. However, the concentration is limited compared to that in disordered LiNi_2/3_Nb_1/3_O_2_ and therefore cationic Ni redox is dominant in Li_1–*y*_Ni_1/2_Mn_1/2_O_2_, as shown in Supporting Figure S12.

### Activation and Destabilization Mechanisms of Anionic Redox for Ni-Based Electrode Materials

**Figure 9:**
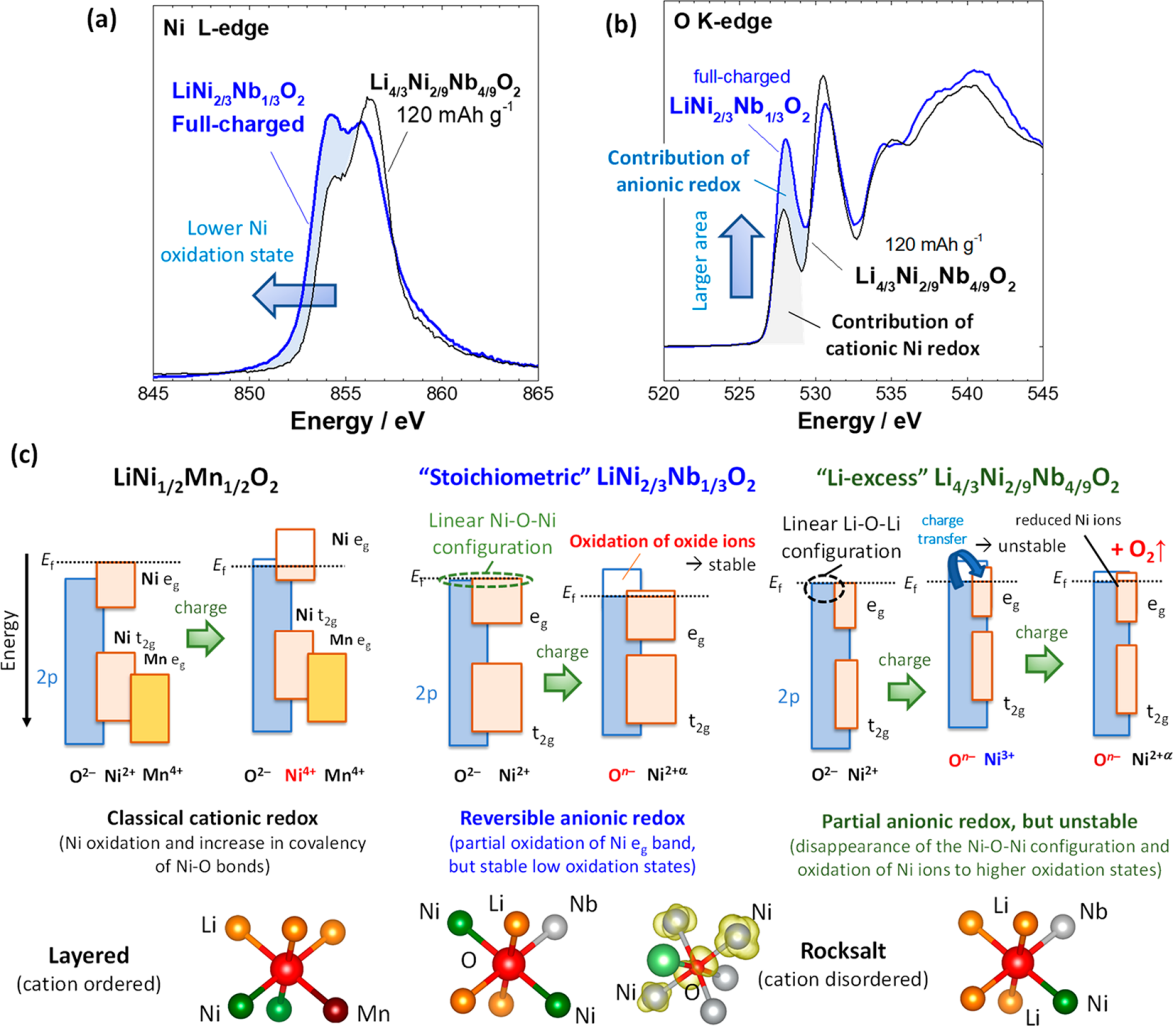
Summary of the activation and stabilization mechanism of anionic redox for Ni-based oxides. Comparison of (a) Ni L edge and (b) O K edge XAS spectra of stoichiometric Li_1–*y*_Ni_2/3_Nb_1/3_O_2_ (charged to 4.8 V) and Li-excess Li_4/3-*y*_Ni_2/9_Nb_4/9_O_2_ (half-charged to 120 mA h g^–1^). The average oxidation state of the Ni ions is clearly lower for the stoichiometric sample in panel a, but a much higher peak intensity is noted at 528 eV in panel b. The appearance of this peak in the O K edge spectrum cannot be explained only by the oxidation of Ni ions, indicating that the formation of the unoccupied state for oxygen is largely responsible for this peak in the stoichiometric sample. (c) Schematic illustrations of cationic and anionic redox processes for a classical layered material LiNi_1/2_Mn_1/2_O_2_, stoichiometric LiNi_2/3_Nb_1/3_O_2_, and Li-excess Li_4/3_Ni_2/9_Nb_4/9_O_2_. The differences in the local environment of O, shown at the bottom, influence the activation and stabilization of anionic redox. For the linear Ni–O–Ni configuration of LiNi_2/3_Nb_1/3_O_2_ with the disordered structure, the contribution of oxygen for charge compensation is large and thus Ni ions retain low oxidation states, which do not destabilize anionic redox. In contrast, the small coordination number of Ni to O for Li_4/3_Ni_2/9_Nb_4/9_O_2_ destabilizes anionic redox because of the formation of unstable Ni ions with higher oxidation states by delithiation. The linear Ni–O–Ni configuration is not formed in layered LiNi_1/2_Mn_1/2_O_2_ and therefore anionic redox is not activated.

In this manuscript, a systematic study of the binary system of Li_3_NbO_4_–NiO has been conducted. On the basis of experimental and theoretical findings, detailed stabilization and destabilization mechanisms of anionic redox for the stoichiometric and Li-excess systems are summarized in Figure 9. In classical layered materials, such as LiNi_1/2_Mn_1/2_O_2_, the Fermi level of the sample consists of mainly Ni e_g_ orbitals. Through oxidation (electrochemical Li extraction), electrons are extracted from the Ni e_g_ orbitals near the Fermi level, corresponding to oxidation from Ni^2+^ to Ni^4+^. The enhancement of covalency with O 2p orbitals after oxidation results in partial charge transfer from O ions to Ni ions (Figure 4b and Supporting Figure S12). Nevertheless, on the basis of changes in the net spin moments of Ni and O obtained by theoretical calculations for the layered materials (Figure 8b and Supporting Figure S23), this system is essentially classified as the classical “cationic” redox process.

For LiNi_2/3_Nb_1/3_O_2_, substitution with Nb^5+^, which has a high ionic character, increases the energy of the O 2p orbitals relative to the Fermi level, leading to the increased accessibility of O redox upon charge and the lower contribution of Ni redox. In addition, Nb substitution triggers structural disordering; thus, the linear Ni–O–Ni configuration with a relatively strong antibonding character is formed near the Fermi level. Therefore, the large contribution of oxygen to charge compensation is achieved in this environment through σ-type interactions with the Ni e_g_ orbitals from both sides of the oxygen 2p orbitals (Figure 7c-1). Note that a similar phenomenon is also known for rocksalt NiO with a higher concentration of the Ni–O–Ni configuration. The band gap of NiO is sandwiched between the filled oxygen 2p band and the empty upper Hubbard band (Ni 3d band).^69^ Additionally, NiO is known as a charge-transfer insulator.^70^ Therefore, the oxidation of NiO (and the formation of Ni_1–*x*_O) induces holes in the O 2p bands near the Fermi level without oxidation of Ni ions, as evidenced by theoretical and experimental XAS studies.^69^ Ni_1–*x*_O is classified as a negative charge-transfer insulator with a narrow band gap, which is formed by the split of the O 2p band.^69^ The partial oxidation of Ni ions also proceeds in Li_1–*y*_Ni_2/3_Nb_1/3_O_2_, as evidenced experimentally in Figure 4a and theoretically in Figure 8a. The partial oxidation of Ni ions possibly influences profile changes of the O K edge spectra. However, the average oxidation state of the Ni ions is lower when compared with thoseof layered materials (Figure 4b and Supporting Figure S13). When the XAS spectra of the fully charged stoichiometric and half-charged Li-excess samples are compared, the oxidation state of the Ni ions is higher for the half-charged Li-excess sample, as shown in Figure 9a. Nevertheless, a much larger peak area at 528 eV was observed in the O K edge XAS spectra of the fully charged stoichiometric sample, as shown in Figure 9b. From this comparison and the results of theoretical study, it was concluded that anionic redox with a σ-type interaction was observed at 528 eV in the O K edge spectra, which was found to have an energy similar to that of oxygen bonded to Ni^3+^ (Ni^4+^) ions (see Figure 9b). For the case of the binary system of Li_3_NbO_4_–LiMnO_2_, as well as other conventional Li_2_MnO_3_-based layered materials, the peak at 528 eV is not visible in the O K edge XAS spectra after charge.^55^ Instead, a new peak appears at 530 eV, and the peak intensity evolves as the charge capacity increases. The origin of this peak has been explained by the formation of π-like bonds between Mn^4+^ (t_2g_^3^) and O 2p orbitals after lithium extraction^25,44^ or the presence of filled π-orbitals for O_2_ molecules.^17,34^ After the oxidation of O in the Li–O–Li environment (essentially a nonbonding state), the anionic redox process is energetically stabilized by electron donation from Mn^4+^ ions, resulting in the rehybridization of the molecular orbitals and the formation of a weak π-type bond between Mn^4+^ and oxygen. On the basis of these differences in the O K edge XAS spectra profile evolution, the nature of the chemical bonds (σ- or π-type interaction with transition metals) is proposed to influence the profiles of the O K edge XAS spectra. Observations similar to those for the Li_3_NbO_4_–NiO system were also noted for the Fe^3+^ systems in which new peaks appear at *ca*. 528 eV, which was also observed for SrFeO_3_ (Figure 4c).^54,55,71^ Although Fe (d^5^ high-spin configuration; t_2g_^3^ e_g_^2^) possibly stabilizes anionic redox through a σ-type interaction, oxygen loss is also a dominant process in this system, as observed in Li_4/3_Fe^3+^_1/3_Sb_1/3_O_2_.^12^

Further enrichment of Li or Nb ions in the structure changes this scenario. Li or Nb enrichment inevitably reduces the Ni ion content, namely decreasing the coordination number of Ni to O. The concentration of oxygen in the linear Ni–O–Ni configuration is also reduced, and the linear Li–O–Li configuration is formed instead. As a result, Ni oxidation is unavoidable upon charge, similar to the Ni-based layered materials. Indeed, the average oxidation state of the Ni ions after charge is higher for the Li-excess phase than the disordered phase, as shown in Figure 9. Oxygen redox also occurs in the linear Li–O–Li configuration. Unstable Ni ions with higher oxidation states destabilize anionic redox and result in charge donation to Ni from O, causing Ni reduction and O_2_ release, *i.e*., reductive coupling.^12,55^ Such Ni reduction upon “charge” is clearly visible in the XAS spectra shown in Figure 4a. The fraction of linear Ni–O–Ni configurations in the structure, which facilitate the stabilization of anionic redox through the formation of σ-type bonds, is too small and therefore oxygen loss cannot be suppressed for the Li-excess phase (Figure 3 and Supporting Figure S16 and S17).

Although the oxygen redox and its stabilization were evidenced for the stoichiometric phase, the reversible capacity is limited to only 85 mAh g^–1^. This fact probably suggests that electrode kinetics in this system are essentially low. Additionally, the oxygen unoccupied state is not itinerant and is probably localized in this structure. Indeed, in contrast to the Ni–Nb system, highly reversible anionic redox and excellent electrode kinetics were achieved for Li_2–*y*_RuO_3_ with an itinerant electron system.^72^ Nevertheless, as shown in Figure 2a, the reversible capacity for Li_12/11_Ni_6/11_Nb_4/11_O_2_ (*x* = 0.40) is further increased because of the increased percolation probability for Li migration in the structure due to Li enrichment.^73^ Additionally, our recent work demonstrates that Li migration kinetics for the stoichiometric phase were drastically increased by the preparation of nanometer-sized samples.^74^ Furthermore, anionic redox is more readily activated for nanometer-sized samples at lower operating temperatures.^75,76^ Note that for Li_6/5_Ni_2/5_Nb_2/5_O_2_(*x* = 0.50) the conversion reaction is clearly present after discharge, which suggests that anionic redox is destabilized in this composition. Because of the lower concentration of the linear Ni–O–Ni configuration in the structure, 40% Ni in the structure is not enough to suppress Ni oxidation and stabilize the unoccupied state of oxygen. Therefore, further increases in the energy density of positive electrode materials with anionic redox are anticipated through the optimization of particle sizes, the chemical composition, and the coordination environment for oxygen.

## Conclusions

The reaction mechanisms of Li_3_NbO_4_–NiO binary oxides as positive electrode materials have been systematically examined, and the stabilization and destabilization mechanisms for anionic redox discussed. Direct evidence is presented for electrochemical anionic redox, which is stabilized through the formation of the linear Ni–O–Ni configuration and σ-type interactions with transition metal e_g_ orbitals. Soft XAS and theoretical studies reveal that reversible anionic redox with a small voltage hysteresis occurs in stoichiometric LiNi_2/3_Nb_1/3_O_2_ with a cation-disordered Li/Ni arrangement. In addition, the importance of crystal structures is also noted. Anionic redox is activated only in the disordered structure, and classical cationic redox is dominant in the ordered and layered structures. Although Li enrichment in the structure, and the formation of the Li–O–Li configuration, was proposed to be the necessary condition to activate anionic redox, careful analysis in this study with different structures/ and compositions reveals that structural disordering and the formation of the linear Ni–O–Ni configuration can also activate anionic redox. Moreover, the enrichment of the Li–O–Li configuration in this Ni-based system results in oxygen loss upon charge because of the loss of the Ni–O–Ni configuration and the formation of unstable Ni ions in the host structure. The activation and stabilization mechanisms of anionic redox found in stoichiometric LiNi_2/3_Nb_1/3_O_2_ with a cation-disordered Li/Ni arrangement cannot be explained by the theory of anionic redox developed in Li-excess Mn-based oxides. Therefore, further research progress on anionic redox in electrode materials with different oxygen local structures is anticipated in the future. These findings provide a new direction for both the design of a class of 4 V positive electrode materials with reversible anionic redox through the engineering of the oxygen coordination environment and the development of high-energy lithium-ion batteries in the future.

## Experimental Methods

### Synthesis of Materials

The target materials, generally with composition *x*Li_3_NbO_4_–(1 – *x*)NiO, were synthesized by the solid-state method. Li_2_CO_3_ (98.5%, Kanto Kagaku), NiCO_3_·Ni(OH)_2_·4H_2_O (Kishida Chemical), and Nb_2_O_5_ (99.9%; Wako Pure Chemical Industries) were mixed, followed by wet ball-milling with methanol at 300 rpm for 6 h. After drying, the obtained powder was pressed into pellets under a pressure of 20 MPa. The obtained pellets were calcined in air at 1000 °C for 48 or 2 h.

A polycrystalline sample of SrFeO_3_ was synthesized via a solid-state reaction under high-pressure conditions. A mixture of stoichiometric amounts of SrCO_3_ and Fe_2_O_3_ was first calcined at 1200 °C for 24 h in air. The obtained calcined powder was sealed in a Pt capsule with an oxidizing agent, KClO_4_, and held at 4.0 GPa and 1000 °C for 30 m before being quenched to room temperature. The pressure was then reduced slowly to ambient. The reacted sample was washed with distilled water to remove the residual KCl and KClO_4_.

### Characterization

X-ray diffraction (XRD) patterns of the samples were collected using an X-ray diffractometer (D2 PHASER, Bruker) equipped with a one-dimensional X-ray detector using Cu Kα radiation generated at 300 W (30 kV and 10 mA) with a Ni filter. Structural analysis was carried out using RIETAN-FP.^77^ Schematic illustrations of crystal structures were drawn using the program VESTA.^78^ Neutron diffraction patterns were collected at BL09 (SPICA) in the Material and Life science Facility (MLF) of the Japan Proton Accelerator Research Complex (J-PARC).^79^ Approximately 1 g of the powder samples was loaded in a cylindrical vanadium can with a 6 mm diameter (PV-6-F, Taiyo Koko Co. Ltd.). Structural analysis was carried out using Z-Rietveld.^80,81^ The particle morphology of the samples was observed using a scanning electron microscope (JCM-6000, JEOL) with an acceleration voltage of 15 keV. Transmission electron microscopy (TEM) was conducted using a JEM-ARM200F (JEOL) microscope operated at 200 keV. The samples were dispersed in dimethyl carbonate and then supported on a copper grid.

For the electrochemical characterization, as-prepared oxides were mixed with acetylene black (AB, HS-100, Denka) and PVdF (no. 1100, Koreha), oxide/AB/PVdF = 80:10:10 wt %, and pasted on aluminum foil (Hosen Corp.) as a current collector. Metallic lithium (Honjo Metal) was used as a negative electrode. The electrolyte solution used was 1.0 mol dm^–3^ LiPF_6_ dissolved in ethylene carbonate (EC)/dimethyl carbonate (DMC) 1:1 v/v (battery-grade, Kishida Chemical). A polyolefin microporous membrane (Celgard 2500) was used as a separator. Two-electrode-type Li cells (Tomcell Japan, type TJ-AC) were assembled in an Ar-filled glovebox. The cells were cycled at a rate of 5 or 10 mA g^–1^ at 50 °C. Sample loading ranged from 6–8 mg cm^–2^, with sample thicknesses of 60–75 μm. The reversible capacity provided in this study was calculated on the basis of the masses of active materials.

Coin cells for *in operando* experiments were constructed from casings with 3 mm diameter holes and a stainless-steel spacer with a 5 mm hole, which allowed the X-rays to be transmitted through the cell. The cells were constructed in an Ar-filled glovebox, and the holes in the casing were sealed using Kapton tape on both the inside and the outside to prevent air exposure. In addition to the electrode in question (LiNi_2/3_Nb_1/3_O_2_ (*x* = 0.33) and Li_4/3_Ni_2/9_Nb_4/9_O_2_ (*x* = 0.67), diameter of 12 mm), the coin cells contained a wave spring, a ∼1 mm thick Li metal plate, and a glass fiber separator with 110 μL of 1.0 M LiPF_6_ in an EC/DMC (1:1 by volume) electrolyte solution (battery-grade, Sigma-Aldrich). The cells were transported in a vacuum sealed bag, and *in operando* experiments were carried out within 48 h of cell construction.

*In operando* synchrotron powder XRD experiments were performed at the Powder Diffraction beamline,^82^ Australian Synchrotron, Clayton, Victoria, Australia. The coin cells were mounted on the beam using a custom-made holder and cycled using a Neware battery tester. During cycling, the cells were heated to 50 °C using a hot air blower, and the temperature was continuously monitored via a thermocouple mounted adjacent to the sample. A nominal X-ray beam energy of 18 keV (λ = 0.68745(2) Å) and beam dimensions 2.0 × 0.5 mm were used. The exact wavelength and the instrumental contribution to the peak broadening or profile were determined by calibration from an XRD pattern collected on a NIST LaB_6_ 660b line profile standard. The XRD data were collected using a Mythen position-sensitive silicon microstrip detector that moved between two positions (0.5° separation, 90 s exposure at each position), with a time resolution of 3.33 min per data set. The data sets were merged and normalized using the program PDViPeR, and Rietveld analysis was carried out with the Fullprof Suite software package.^83^

An online mass electrochemical mass spectrometry (OEMS) system was used as described in a previous work.^50^ The custom-designed OEMS cell (Tomcell Japan) was comprised of electrode materials, lithium foil as the negative electrode, and 1 M LiPF_6_ in a mixture of EC and DMC as the electrolyte solution. The assembled OEMS cell was rested in an Ar-filled glovebox for 2 h and linked to the OEMS apparatus in a constant-temperature container, maintaining a temperature of 25 °C. The OEMS cell was purged with Ar (99.9999%) for a total of 10 min to test the system. Cell pressure (16–18 psi) and the mass spectrum were also monitored at an open-circuit voltage at room temperature. The CO_2_ gas evolution from DMC evaporation stabilized after 3 h and was then set as the background. The galvanostatic charging process was carried out at a specific current of 10 mA g^–1^. Gas analysis was conducted after the collection of gas products every 1 h.

Hard X-ray absorption spectroscopy (XAS) at the Ni K edge was performed at beamline BL-12C of the Photon Factory synchrotron source in Japan. Hard X-ray absorption spectra were collected with a silicon monochromator in the transmission mode. The intensities of the incident and transmitted X-rays were measured using an ionization chamber at room temperature. The composite electrodes were rinsed with dimethyl carbonate and sealed in a water-resistant polymer film (Kuraray, type EF-E) in an Ar-filled glovebox.

Soft X-ray absorption (XAS) spectra were collected at the BL-11 (O K edge and metal L_II,III_ edges) beamline in the synchrotron facility of Ritsumeikan University (Synchrotron Radiation Center). The absorption spectra were collected in the fluorescence yield mode. Similar to the hard XAS measurement, the samples were prepared in an Ar-filled glovebox. The thus-prepared samples were placed on the spectrometer using a laboratory-made transfer vessel without air exposure.

First-principles calculations based on density functional theory (DFT)^84^ were performed using the Vienna *ab initio* simulation package (VASP)^85,86^ with both the modified Perdew–Burke–Ernzerhof generalized gradient approximation for a solid (PBEsol-GGA)^87,88^ and the projector-augmented wave (PAW) method.^89^ On-site Coulomb correction (GGA + *U*) was included for localized electronic states, and the *U* value for the Ni 3d states was set to 6.0 eV in accordance with the literature.^90^ The Heyd–Scuseria–Ernzerhof (HSE06)^91,92^ hybrid functional was also used to correct the self-interaction errors in accordance with the literature^41,93^ and to obtain precise electronic structures. A kinetic cutoff energy was set to 750 eV, and *k*-point meshes were sampled such that the product of meshes and atoms was approximately 800. For example, 2 × 3 × 2 *k*-point meshes were employed for 18 formula units of Li_1–*y*_Ni_2/3_Nb_1/3_O_2_ (*x* = 0.33). These settings were found by convergence tests (<3 meV per Li_1–*y*_Ni_2/3_Nb_1/3_O_2_). Note that mesh densities were increased by a factor of approximately 1.5–2.0 when the density of states (DOS) profiles were computed for visualization purposes.

### Safety Statement

No unexpected or unusually high safety hazards were encountered.

## Supporting Information Available

Refined parameters and fitting results; structural parameters used for the theoretical calcultion; particle morphologies; TEM images; electron diffraction patterns; comparison of XRD patterns and SEM images for LiNi_2/3_Nb_1/3_O_2_ synthesized by different conditions; capacity retention of LiNi_2/3_Nb_1/3_O_2_ and Li_4/3_Ni_2/9_Nb_4/9_O_2_; initial charge–discharge profiles; *ex situ* XRD pattterns; *in operando* synchrotron XRD contour map; structural parameters; EXAFS spectra; Nb K edge, Ni L edge, and O K edge spectra; HR-TEM images; schematic illustration of hypothetical layered-type LiNb_1/3_Ni_2/3_O_2_; and calculated net spin moments (PDF)
