# Supplementary material for: Unexpectedly Large Contribution of Oxygen to Charge Compensation Triggered by Structural Disordering: Detailed Experimental and Theoretical Study on a Li3NbO4–NiO Binary System
Source: ACS Cent Sci. 2022 May 23;8(6):775–94. doi: 10.1021/acscentsci.2c00238 (PMC9228563; doi:10.1021/acscentsci.2c00238)
Supplement: Supplementary file 1 — oc2c00238_si_001.pdf [file oc2c00238_si_001.pdf]

Supporting Information for

Unexpectedly Large Contribution of Oxygen on Charge Compensation Triggered by Structural Disordering: Detailed Experimental and Theoretical Study on Li_3NbO_4-NiO Binary System

Ryutaro Fukuma[1†], Maho Harada,[2†] Wenwen Zhao[1], Miho Sawamura[1], Yusuke Noda[3, 4], Masanobu Nakayama[2, 3, 5], Masato Goto[6], Daisuke Kan[6], Yuichi Shimakawa[6], Masao Yonemura[7, 8], Naohiro Ikeda,[9] Ryuta Watanuki[5, 9], Henrik L. Andersen[10], Anita M. D'Angelo[11], Neeraj Sharma[10], Jiwon Park[12], Hye Ryung Byon[12], Sayuri Fukuyama[13], Zhenji Han[13], Hitoshi Fukumitsu[13], Martin Schulz-Dobrick[13], Keisuke Yamanaka[14], Hirona Yamagishi[14], Toshiaki Ohta[14], and Naoaki Yabuuchi[5, 9, 15]*

[1]Department of Applied Chemistry, Tokyo Denki University, 5 Senju Asahi-cho, Adachi-ku, Tokyo 120-8551, Japan

[2]Frontier Research Institute for Materials Science (FRIMS), Nagoya Institute of Technology, Gokiso-cho, Showa-ku, Nagoya, Aichi 466-8555, Japan

[3]GREEN & MaDiS/CMi[2], National Institute of Materials Science (NIMS), 1-2-1, Sengen, Tsukuba, Ibaraki 305-0047, Japan

[4]Department of Information and Communication Engineering, Okayama Prefectural University, 111 Kuboki, Soja, Okayama 719-1197, Japan

[5]Elements Strategy Initiative for Catalysts and Batteries, Kyoto University, f1-30 Goryo-Ohara, Nishikyo-ku, Kyoto 615-8245, Japan

[6]Institute for Chemical Research, Kyoto University, Uji, Kyoto 611-0011, Japan

[7]Institute of Materials Structure Science, High Energy Accelerator Research Organization, Tsukuba, Ibaraki, 305-0801, Japan

[8]Department of Materials Structure Science, Sokendai (The Graduate University for Advanced Studies), 203-1 Shirakata, Tokai, Ibaraki 319-1106, Japan

[9]Department of Chemistry and Life Science, Yokohama National University, 79-5 Tokiwadai, Hodogaya-ku, Yokohama, Kanagawa 240-8501, Japan

[10]School of Chemistry, UNSW Australia, Sydney, NSW 2052, Australia

[11]Australian Synchrotron, Clayton, VIC 3168, Australia

[12]Department of Chemistry, Korea Advanced Institute of Science and Technology (KAIST), and KAIST Institute for NanoCentury, 291 Daehak-ro, Yuseong-gu, Daejeon 34141, Republic of Korea

[13]Battery Materials Laboratory, BASF Japan Ltd., 7-1-13 Doi-cho, Amagasaki, Hyogo 660-0083, Japan

[14]SR Center, Ritsumeikan University, 1-1-1 Noji-Higashi, Kusatsu, Shiga 525-8577, Japan

[15]Advanced Chemical Energy Research Center, Yokohama National University, 79-5 Tokiwadai, Hodogaya-ku, Yokohama, Kanagawa 240-8501, Japan

† These authors contributed equally to this work.

*corresponding author, e-mail: yabuuchi-naoaki-pw@ynu.ac.jp

Supporting Results and Discussion

S1. Structural changes of stoichiometric $Li_{1-y}Ni_{2/3}Nb_{1/3}O_2$ *and Li-excess* $Li_{4/3-y}Ni_{2/9}Nb_{4/9}O_2$

Ex-situ XRD patterns of the stoichiometric $LiNi_{2/3}Nb_{1/3}O_2$ and Li-excess $Li_{4/3}Ni_{2/9}Nb_{4/9}O_2$ samples during the first cycle were collected (**Supporting Figure S8**). Highly reversible changes in the crystal structure are noted in $Li_yNi_{2/3}Nb_{1/3}O_2$. Diffraction lines shift toward higher diffraction angles upon charge to 4.8 V. The unit cell volume is reduced from 880.7 to 866.0 Å^3 from the as-prepared to fully charged sample. After discharge to 1.5 V, identical diffraction lines compared to the as-prepared sample are noted with no increase in peak width. From this observation, it is concluded that Li extraction/re-insertion proceeds through a reversible topotactic reaction without oxygen loss. For the Li-excess system, a clearly different trend is found. The original highly crystalline phase is lost on charge and significant peak broadening is evidenced during charge. After full discharge to 1.5 V, the peak width narrows relative to the charged state but it is still relatively broad compared to the as-prepared sample. This finding suggests that irreversible structural changes occur in the Li-excess system, probably associated with oxygen loss on charge.

Supporting Figure 9a shows the background subtracted and normalized *in operando* XRD contour map of the $LiNi_{2/3}Nb_{1/3}O_2$ ($x = 0.33$) sample. As seen in the figure, no additional crystalline phases appear during cycling. In addition, little-to-no structural changes, *i.e.* changes in peak positions and relative reflection intensities, are observed as seen in the enhancements of selected 2θ-regions in

Figure 3a. Sequential Rietveld refinements of the data were carried out based on the structure of $LiNi_{2/3}Nb_{1/3}O_2$ adopting orthorhombic symmetry with the space group *Fddd*. Reliable refinement of atomic structures in the *operando* data was not deemed feasible and the atomic structure was consequently fixed to the structure obtained from the neutron diffraction data (**Table S1**) during the sequential refinements. The lattice parameters, scale-factor, zero-shift, and background (Chebychev polynomial) were refined. The contribution from the aluminum foil was accounted for by implementing an aluminum phase (space group *Fm*-3*m*) with a high degree of preferred orientation in the modelling. No additional crystalline phases were observed in the data. The quantitative structural analysis was limited to the relative changes in lattice parameters (which do not rely on peak intensities) and relative variations in the scale factor (see **Supporting Figure 9b**). The scale factor encompasses 2θ-independent contributions, such as incident beam flux, instrumental geometry, detector efficiency, sample volume, *etc*. Assuming the instrumental contributions to be constant in time, observed variations in the refined scale factor of a given phase will be proportional to changes in the amount of coherently scattering material of the given phase. For the $LiNi_{2/3}Nb_{1/3}O_2$ sample, a small continuous increase (~10%) in the scale factor over the entire two electrochemical cycles is observed (**Supporting Figure S9 b-1**). No significant changes are observed in the unit cell parameters, suggesting highly reversible structural changes on electrochemical cycles, which is consistent with the *ex-situ* XRD data. It should be noted that the *ex-situ* and *operando* experiments

were run at different current rates.

Supporting Figure S10a shows the background subtracted and normalized *in operando* XRD contour map of the $Li_{4/3}Ni_{2/9}Nb_{4/9}O_2$ (x = 0.67) sample. As seen in the figure, no major additional crystalline phases are formed during cycling. However, closer inspection of the reflections shows a gradual decrease in the main peak intensities, which is associated with the appearance of a shoulder on the left side (lower 2θ) of the peaks (see **Figure 3a**). The shoulder is observed for all reflections, indicating the formation of an isostructural compound with a larger unit cell, which is expected to be a delithiated (or partially delithiated) disordered rocksalt phase formed upon charge. Notably, the *in operando* XRD study reveals that the degradation of the main phase is seemingly irreversible as it does not reform upon discharge. **Supporting Figure S10b** shows an enhancement of the (200) and (220) reflections at selected times throughout the two electrochemical cycles. Closer inspection of the data also reveals that a more complex series of structural transitions takes place, in particular during the initial charge, where the peaks initially shift to higher 2θ values (unit cell decrease) and subsequently back again along with the appearance of a broad shoulder at lower 2θ. A decrease in the overall combined intensity of the reflections (~25%) is also observed during the first charge (see **Supporting Figure S10b** top left). Sequential Rietveld refinements of the data were carried out based on the disordered rock salt structure of $Li_{4/3-y}Ni_{2/9}Nb_{4/9}O_2$ (space group Fm-$3m$). Again, reliable refinement of atomic structure in the *operando* data was not deemed feasible and the

atomic structure was fixed to the structure obtained from the neutron diffraction data (**Table S2**) during the sequential refinements. Based on the observation of peak shoulders above, an additional delithiated phase (space group *Fm*-3*m*) was implemented in the modelling. Lattice parameters, scale-factor, zero-shift, background (Chebychev polynomial) and one Lorentzian profile parameter associated with isotropic microstrain per phase were refined. The contribution from the aluminum foil was accounted for by implementing an aluminum phase (space group *Fm*-3*m*) with a high degree of preferred orientation in the modelling. Representative Rietveld fits at different levels of charge are shown in **Supporting Figure S10c**. Here, the data has been modelled by the two endmember phases, *i.e.* the original disordered rock salt structure of $Li_{4/3}Ni_{2/9}Nb_{4/9}O_2$ and a delithiated disordered rock salt structure. However, the asymmetrical peak shapes likely arise from a non-trivial distribution of lattice parameters caused by gradual extraction of various amounts of Li from the structure and thus non-uniform Li distributions in certain parts of the sample. The implementation of a third highly strained (partially delithiated) rock salt phase in the refinement did not give substantially better fit and furthermore destabilized the refinement due to parameter correlations (see **Supporting Figure S10d**). The fits to the peak profiles were not entirely robust, and care must be taken not to draw false conclusions based on the Rietveld refined parameters. The refined scale factors suggest a continuous decrease in the amount of the main $Li_{4/3}Ni_{2/9}Nb_{4/9}O_2$ phase along with a concurrent increase in the secondary range of delithiated phases, which confirms the gradual

transformation of the main phase (also see **Figure 3a**). The original phase does not reform but rather continues to diminish during discharge indicating an irreversible degradation. This also supports the observations from *ex-situ* data.

S2. Determination of cation arrangements for $LiNb_{1/3}Ni_{2/3}O_2$ and Li arrangements for delithiated $Li_{1-x}Nb_{1/3}Ni_{2/3}O_2$ by genetic algorithm (GA) approach

For the determination of cation arrangements by GA approach, firstly, arbitrary cationic arrangements in fully lithiated compounds (x = 0.00) are regarded as ternary strings, consisting of three labels: 0 (Li), 1 (Nb), and 2 (Ni). Likewise, binary strings of 0 (Li) and 1 (vacancy) are considered for delithiated samples ($x > 0$). In each combinatorial optimization, we prepare first 20 configurations (ternary/binary strings), where all labels are arranged randomly in the first generation, and evaluate total energies. Twelve low energy configurations among 20 candidates (60%) are selected as survivors and their structural features are succeeded to the next generation by the following four processes: (1) three best structures are succeeded as they are, (2) eight new structures are generated by a two-point crossover technique, (3) another eight are generated by uniform crossover technique with a 50% probability, and (4) the others (*i.e.* four structures) are generated by a mutation technique with 2% probability. To preserve the composition, we used a permutation encoding technique for the above binary or ternary string representations. The generated structure is selected

not to be consistent with the previously calculated configuration (a random arrangement is chosen, in the case above genetic operation cannot produce a new structure). By repeating this procedure, the lowest energy configuration is determined heuristically.

S3. Voltage estimation on hypothetical layered-type $LiNb_{1/3}Ni_{2/3}O_2$ and Li arrangements for delithiated $Li_{1-y}Nb_{1/3}Ni_{2/3}O_2$

For the purpose of comparison, variations of electronic structures during the delithiation process in the hypothetical layered-type $Li_{1-x}Nb_{1/3}Ni_{2/3}O_2$ are examined by first-principles calculations using the HSE06 hybrid functional. **Supporting Figure S23a** displays cation arrangements of hypothetical layered-type $LiNb_{1/3}Ni_{2/3}O_2$ in which Li and transition metals ions (Ni and Nb) stack alternately along *c*-axis, and Ni and Nb ions are assumed to form a honeycomb arrangement in the *a*-*b* plane as seen in Li_2MnO_3. Therefore, the formation of the linear Ni-O-Ni environment is eliminated in this model. Total electronic energies of symmetrically distinct Li/vacancy arrangements for delithiated phases, $Li_{1-y}Nb_{1/3}Ni_{2/3}O_2$, are computed and corresponding voltage profiles are obtained using lowest energies for each composition as shown in **Supporting Figure S23b**. Note that no oxygen dimers are visible over the entire range of the composition y ($0 \leq y \leq 1$ in $Li_{1-y}Nb_{1/3}Ni_{2/3}O_2$), as the minimum bond distance between two oxygen atoms is 2.49 Å (see **Supporting Figure S20**). The calculated voltages are almost constant ranging from ~4.3 to 4.5 V at $y < 2/3$, and at $y > 2/3$ abruptly increase in the voltage

reaching to > 5V.

Supporting Tables and Figures

Table S1. Refined structural parameters of $LiNi_{2/3}Nb_{1/3}O_2$ (x = 0.33) synthesized at 1000 °C for 48 h by the Rietveld method. For stoichiometric $LiNi_{2/3}Nb_{1/3}O_2$, refined results reveal perfect ordering for Nb ions at 8*a* site. However, partial cation mixing between Li and Ni ions at 16*g* and 8*b* sites is also noted, and Li ions preferentially occupy the 8*b* site. Ordering of Nb ions in the structure results in the appearance of additional diffraction lines and leads to distortion of the orthorhombic lattice.

Atom	Site	Occupancy	*x*	*y*	*z*	*B* (Å^2)
Li	16*g*	0.576	0.125	0.125	0.292(3)	0.4
Ni	16*g*	0.424	0.125	0.125	0.292(3)	0.4
Li	16*g*	0.599	0.125	0.625	0.287(4)	0.4
Ni	16*g*	0.401	0.125	0.625	0.287(4)	0.4
Li	8*b*	0.649	0.125	0.625	0.125	0.6
Ni	8*b*	0.351	0.125	0.625	0.125	0.6
Nb	8*a*	1.0	0.125	0.125	0.125	0.3
O	16*f*	1.0	0.125	0.370 (2)	0.125	1.2
O	32*h*	1.0	0.122(2)	0.374 (2)	0.291 (4)	0.8

Space group; *Fddd*, *a* = 5.9365 (1) Å, *b* = 8.4105 (1) Å, *c* =17.8136 (2) Å

R_{wp} = 5.72% and R_B = 5.79%.

Table S2. Refined structural parameters of $Li_{4/3}Ni_{2/9}Nb_{4/9}O_2$ (x = 0.67) synthesized at 1000 °C for 48 h by the Rietveld method. For Li-excess $Li_{4/3}Ni_{2/9}Nb_{4/9}O_2$, all cations, Li, Ni and Nb ions, are located at the same 4*a* site, forming a completely disordered rocksalt structure, and $Li_{4/3}Ni_{2/9}Nb_{4/9}O_2$ is isostructural with NiO.

Atom	Site	Occupancy	*x*	*y*	*z*	*B* (Å^2)
Li	4*a*	0.670	0	0	0	0.4
Ni	4*a*	0.111	0	0	0	0.4
Nb	4*a*	0.219	0	0	0	0.4
O	4*b*	1.0	0.5	0.5	0.5	1.0

Space group; *Fm*-3*m*, *a* = 4.2009 (3) Å

R_{wp} = 4.91% and R_B = 1.34%

Table S3. Refined structural parameters of $SrFeO_3$ by the Rietveld method.

atom	site	*g*	*x*	*y*	*z*	*B* (Å^2)
Sr	1*b*	0.978(1)	0.5	0.5	0.5	0.445
Fe	1*a*	0.980(3)	0	0	0	0.170
O	3*d*	0.988(6)	0	0	0.5	0.563

Space group; *Pm*-3*m*, a = 3.5809(9) Å

R_{wp} = 8.81% and R_B = 7.63%

Table S4. Refined structural parameters of $LaFeO_3$ by the Rietveld method.

atom	site	*g*	*x*	*y*	*z*	*B* (Å^2)
La	4*c*	0.9631	0.0289(5)	0.25	-0.00769	0.55
Fe	4*b*	1	0	0	0.5	0.56
O1	4*c*	0.9659	0.4859(6)	0.25	0.0762(9)	2.52(5)
O2	8*d*	0.9827	0.2839(2)	0.0316(8)	0.7175(2)	0.879

Space group; *Pnma*, *a* = 5.5437(9) Å, *b* = 7.8253 Å, and *c* = 5.5348(6) Å

R_{wp} = 7.63% and R_B = 6.49%

Table S5. The GA optimized ionic arrangement of the computational supercell model for $Li_{18}Ni_{12}Nb_6O_{36}$ ($LiNi_{2/3}Nb_{1/3}O_2$). Lattice parameters for the supercell are (*a, b, c, α, β, γ*) = (8.90954 Å, 7.85748 Å, 9.84987 Å, 83.4563°, 81.3293°, 79.1066°). This structure model is input for structural relaxation in first-principles calculations.

Atom	Label	*x*	*y*	*z*
Li	Li1	0	0.25	0.75
Li	Li2	0	0.75	0.25
Li	Li3	0.11111	0.08333	0.25
Li	Li4	0.11111	0.33333	0
Li	Li5	0.22222	0.16667	0.5
Li	Li6	0.22222	0.41667	0.25
Li	Li7	0.22222	0.91667	0.75
Li	Li8	0.33333	0.75	0.25
Li	Li9	0.44444	0.08333	0.25

Li	Li10	0.55556	0.66667	0
Li	Li11	0.55556	0.91667	0.75
Li	Li12	0.66667	0.25	0.75
Li	Li13	0.77778	0.08333	0.25
Li	Li14	0.77778	0.33333	0
Li	Li15	0.77778	0.83333	0.5
Li	Li16	0.88889	0.16667	0.5
Li	Li17	0.88889	0.66667	0
Li	Li18	0.88889	0.91667	0.75
Nb	Nb1	0	0	0
Nb	Nb2	0.11111	0.83333	0.5
Nb	Nb3	0.33333	0	0
Nb	Nb4	0.55556	0.16667	0.5
Nb	Nb5	0.77778	0.58333	0.75
Nb	Nb6	0.88889	0.41667	0.25
Ni	Ni1	0	0.5	0.5
Ni	Ni2	0.11111	0.58333	0.75
Ni	Ni3	0.22222	0.66667	0
Ni	Ni4	0.33333	0.25	0.75
Ni	Ni5	0.33333	0.5	0.5
Ni	Ni6	0.44444	0.33333	0
Ni	Ni7	0.44444	0.58333	0.75
Ni	Ni8	0.44444	0.83333	0.5
Ni	Ni9	0.55556	0.41667	0.25
Ni	Ni10	0.66667	0	0
Ni	Ni11	0.66667	0.5	0.5
Ni	Ni12	0.66667	0.75	0.25
O	O1	0.05556	0.04167	0.625
O	O2	0.05556	0.29167	0.375
O	O3	0.05556	0.54167	0.125
O	O4	0.05556	0.79167	0.875
O	O5	0.16667	0.125	0.875
O	O6	0.16667	0.375	0.625
O	O7	0.16667	0.625	0.375
O	O8	0.16667	0.875	0.125
O	O9	0.27778	0.20833	0.125
O	O10	0.27778	0.45833	0.875

O	O11	0.27778	0.70833	0.625
O	O12	0.27778	0.95833	0.375
O	O13	0.38889	0.04167	0.625
O	O14	0.38889	0.29167	0.375
O	O15	0.38889	0.54167	0.125
O	O16	0.38889	0.79167	0.875
O	O17	0.5	0.125	0.875
O	O18	0.5	0.375	0.625
O	O19	0.5	0.625	0.375
O	O20	0.5	0.875	0.125
O	O21	0.61111	0.20833	0.125
O	O22	0.61111	0.45833	0.875
O	O23	0.61111	0.70833	0.625
O	O24	0.61111	0.95833	0.375
O	O25	0.72222	0.04167	0.625
O	O26	0.72222	0.29167	0.375
O	O27	0.72222	0.54167	0.125
O	O28	0.72222	0.79167	0.875
O	O29	0.83333	0.125	0.875
O	O30	0.83333	0.375	0.625
O	O31	0.83333	0.625	0.375
O	O32	0.83333	0.875	0.125
O	O33	0.94444	0.20833	0.125
O	O34	0.94444	0.45833	0.875
O	O35	0.94444	0.70833	0.625
O	O36	0.94444	0.95833	0.375

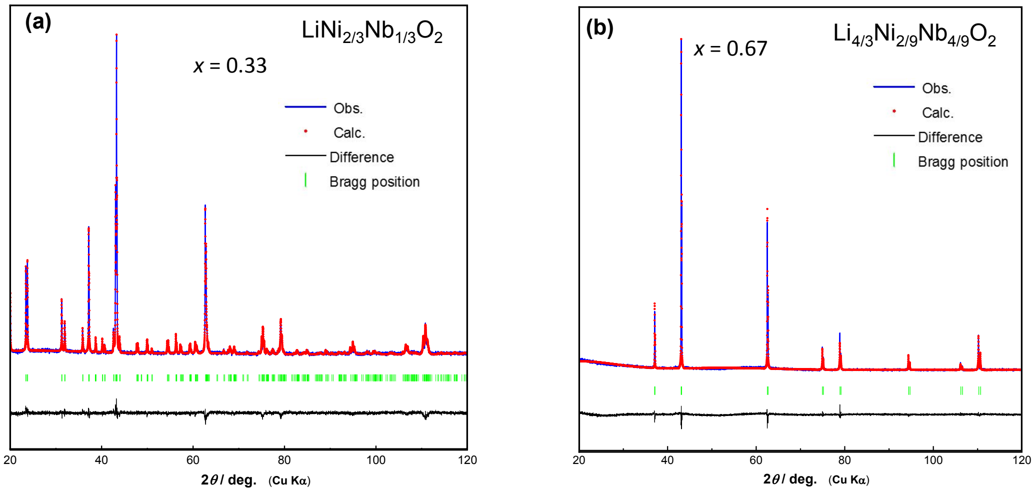

Figure S1. Rietveld-refined fits of $LiNi_{2/3}Nb_{1/3}O_2$ ($x = 0.33$) and $Li_{4/3}Ni_{2/9}Nb_{4/9}O_2$ ($x = 0.67$) samples synthesized at 1000 °C for 48 h.

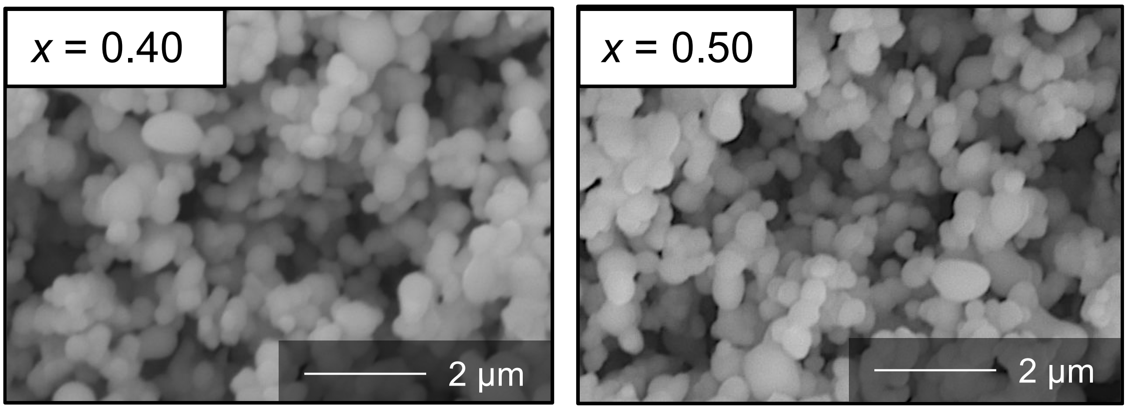

Figure S2. SEM images of $Li_{12/11}Ni_{6/11}Nb_{4/11}O_2$ ($x = 0.40$) and $Li_{6/5}Ni_{2/5}Nb_{2/5}O_2$ ($x = 0.50$) samples synthesized at 1000 °C for 2 h.

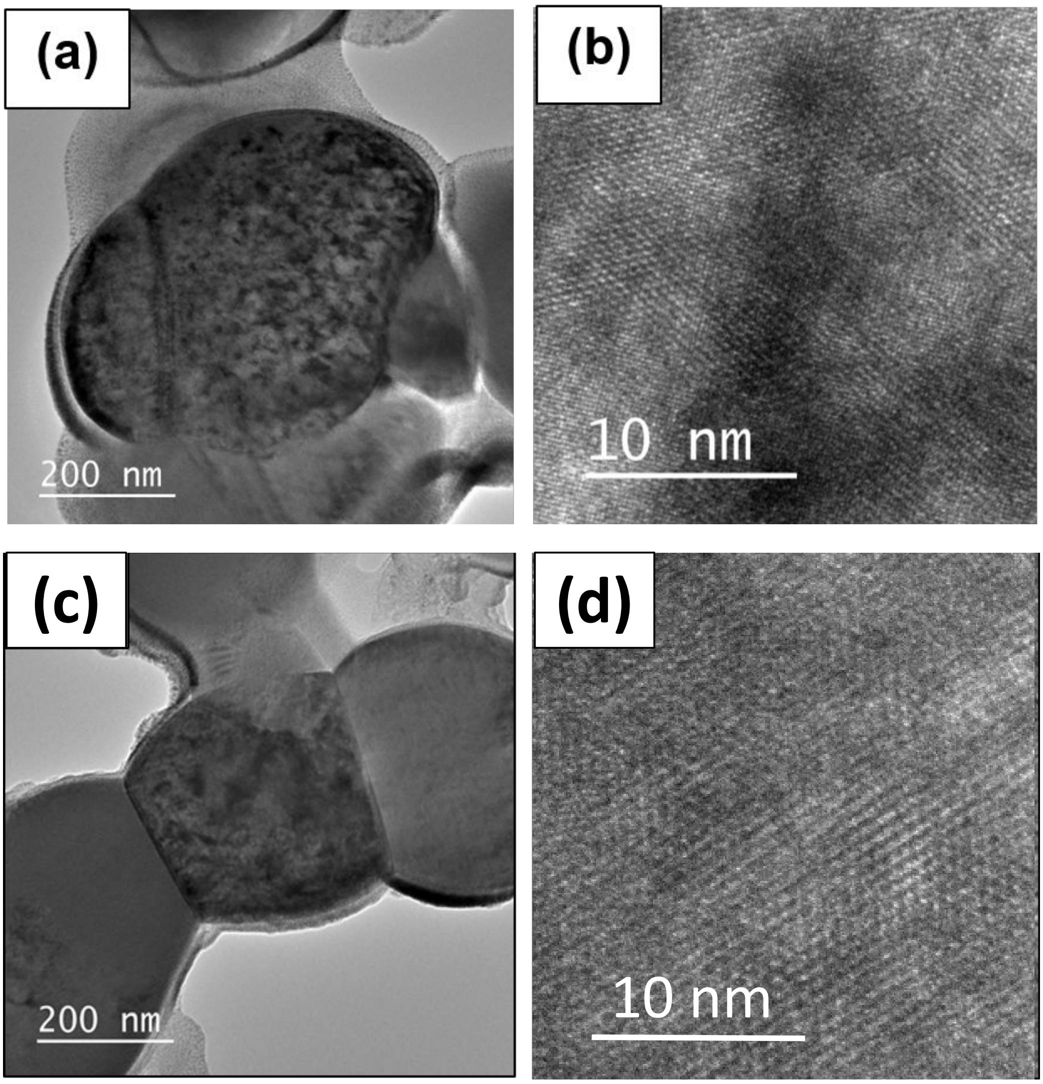

Figure S3. TEM images of (a, b) as-prepared stoichiometric $LiNi_{2/3}Nb_{1/3}O_2$ (x = 0.33) and (c, d) Li-excess $Li_{4/3}Ni_{2/9}Nb_{4/9}O_2$ (x = 0.67).

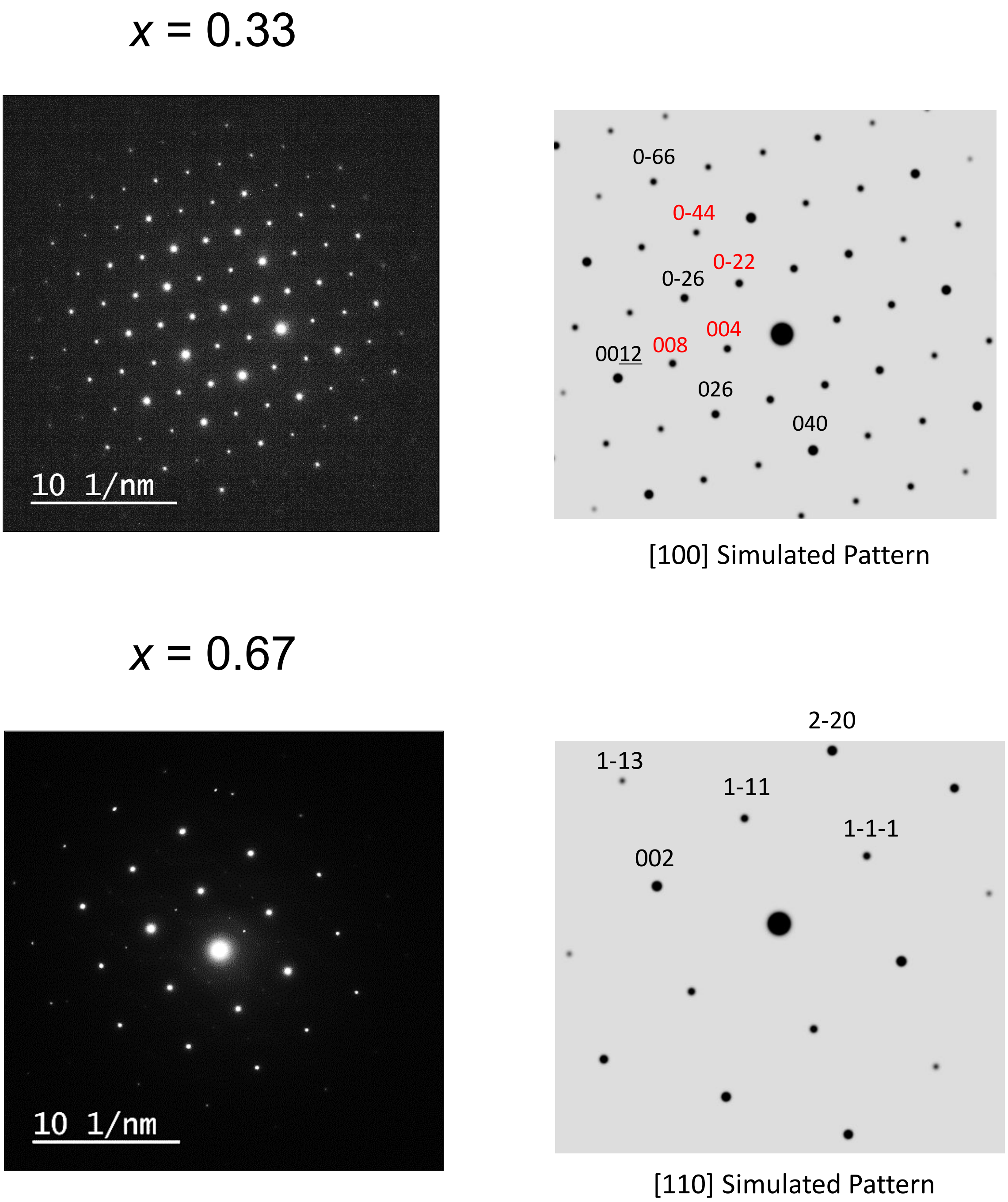

Figure S4. Comparison of observed and simulated ED patterns for the samples of $x = 0.33$ and 0.67. Lattice points indexed with red color correspond to superlattice reflections on the basis of the cation-disordered rock salt phase.

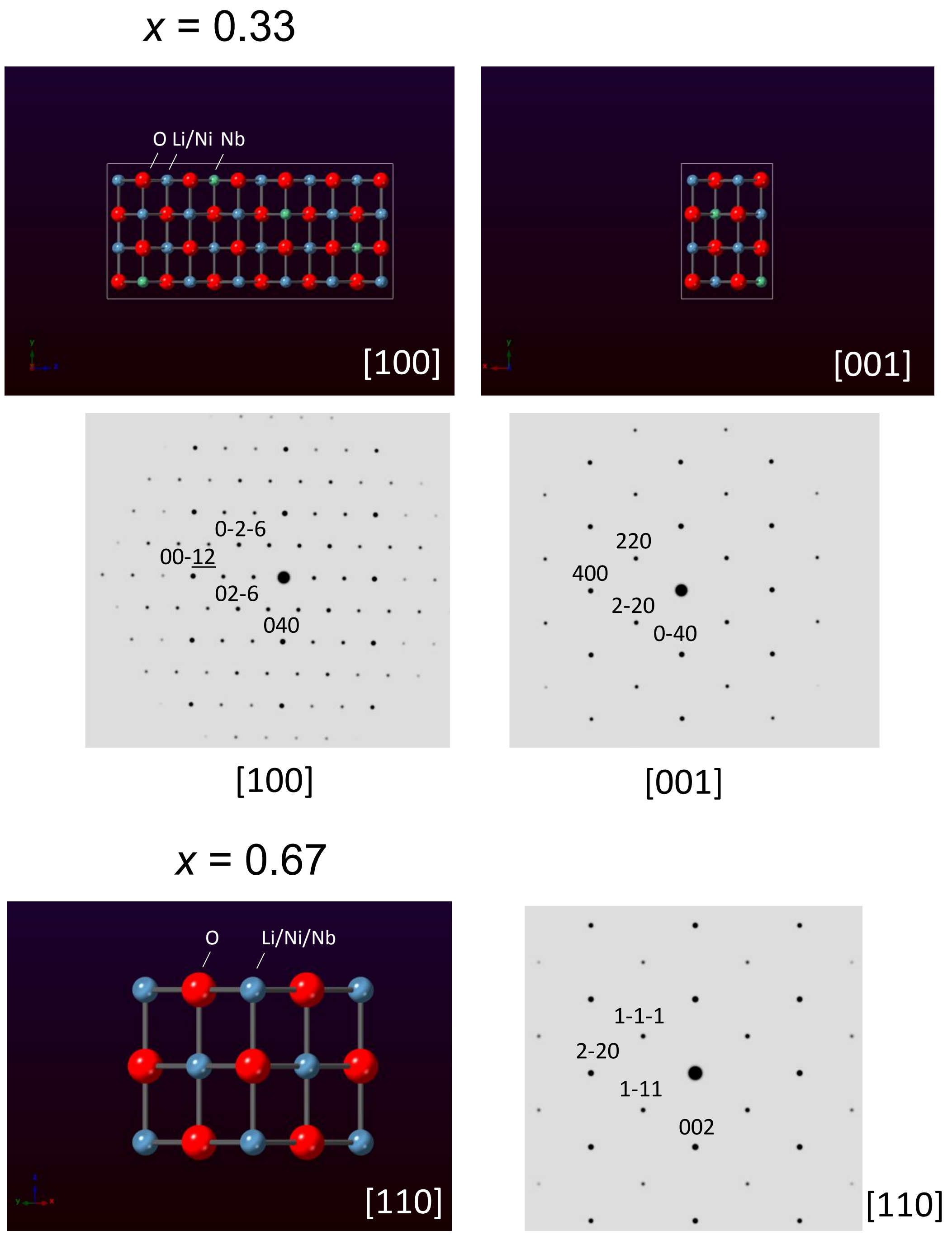

Figure S5. Simulated electron diffraction patterns of the samples of x = 0.33 and 0.67. For the stoichiometric sample (x = 0.33), the superlattice reflections are only observed along [100], and not observed for [001] because the unit cell length is three times larger than the parent rock salt phase in

the *c*-axis direction.

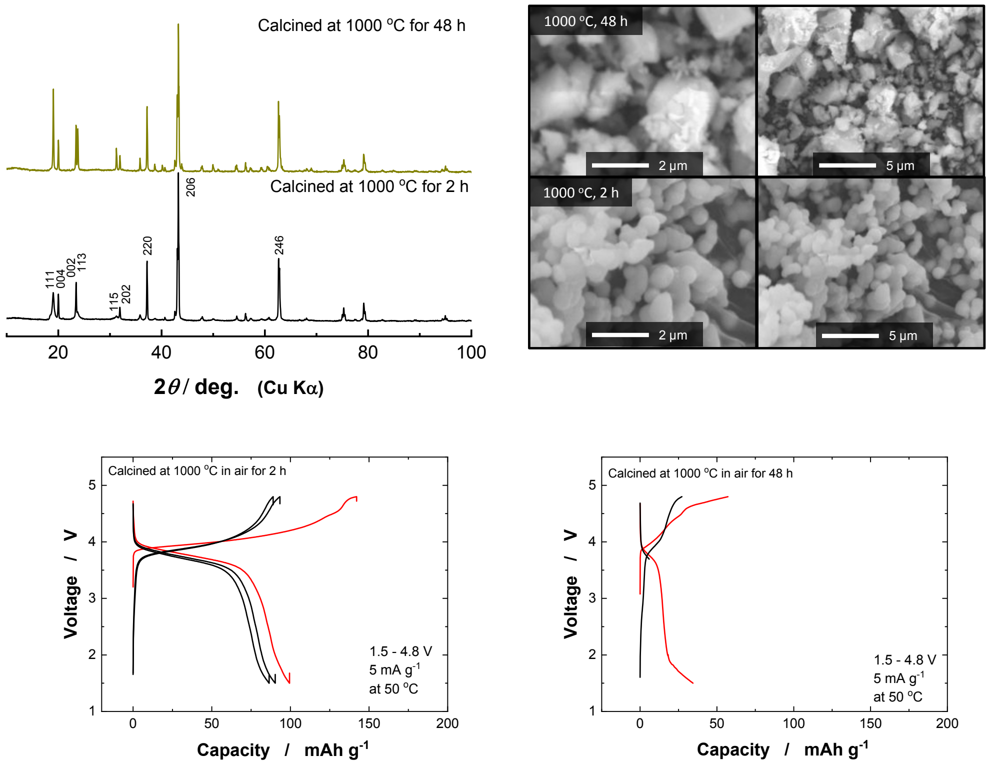

Figure S6. Comparison of XRD patterns, SEM images, and electrode performance of $LiNi_{2/3}Nb_{1/3}O_2$ synthesized at 1000 °C for 48 and 2 h. Synthesis with longer time results in higher degree of cation ordering, but similar cation ordering is also noted for the sample synthesized for 2 h. Moreover, much better electrode performance is observed for the sample synthesized for 2 h, likely associated with particle size differences between the samples.

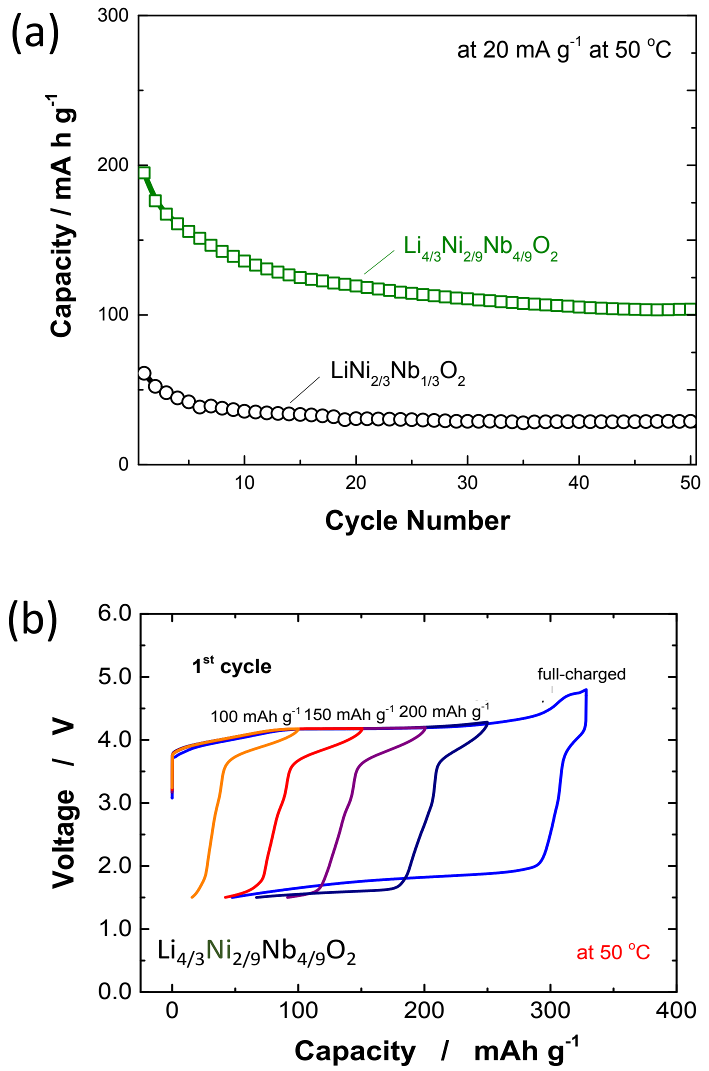

Figure S7. (a) Capacity retention of $LiNi_{2/3}Nb_{1/3}O_2$ and $Li_{4/3}Ni_{2/9}Nb_{4/9}O_2$ at a rate of 20 mA g^{-1} at 50 ^{o}C, and (b) initial charge/discharge profiles of $Li_{4/3}Ni_{2/9}Nb_{4/9}O_2$ at a rate of 5 mA g^{-1} with different charge capacities.

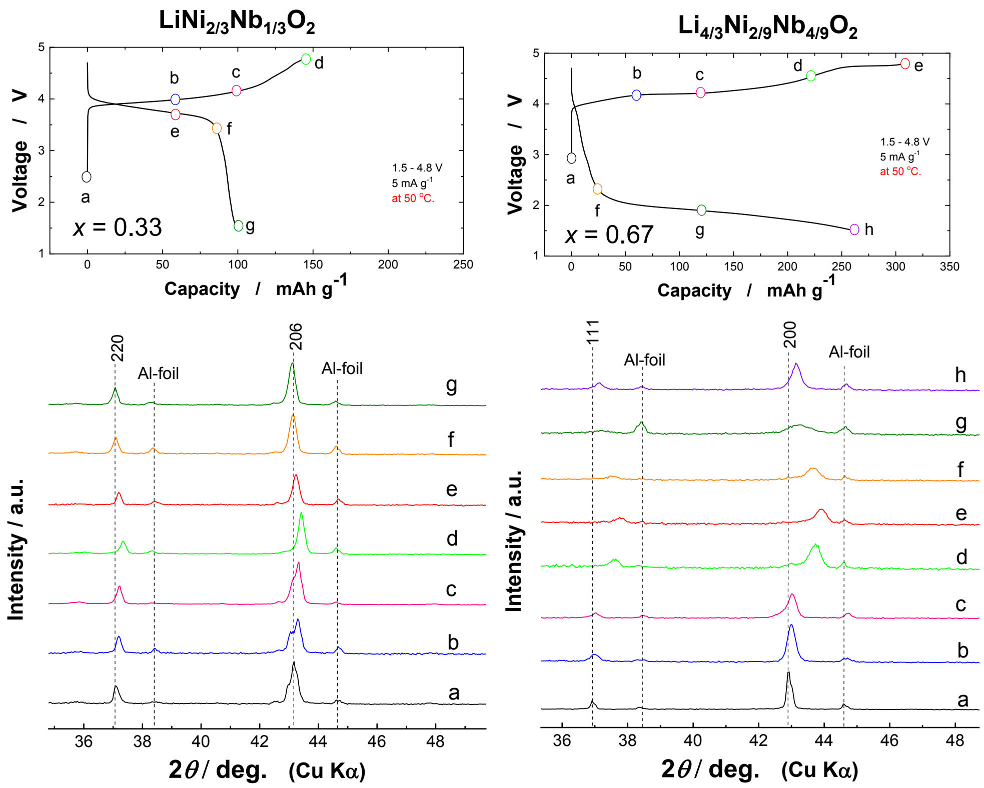

Figure S8. *Ex-situ* XRD patterns of $LiNi_{2/3}Nb_{1/3}O_2$ (x = 0.33) and $Li_{4/3}Ni_{2/9}Nb_{4/9}O_2$ (x = 0.67) samples during the first electrochemical cycle.

(a)

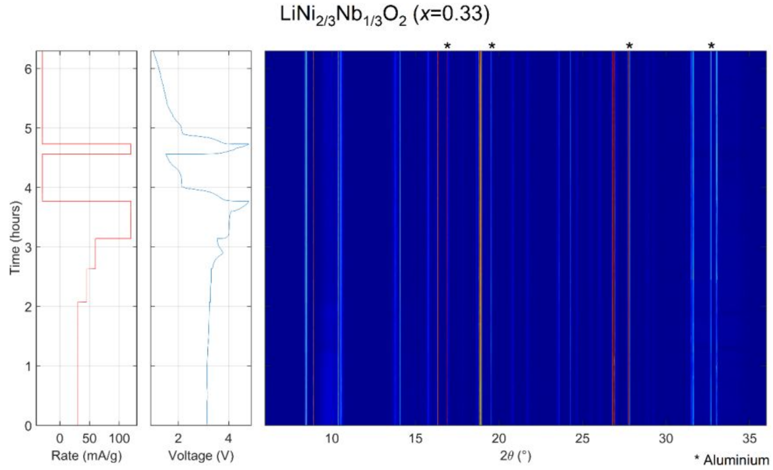

(b)

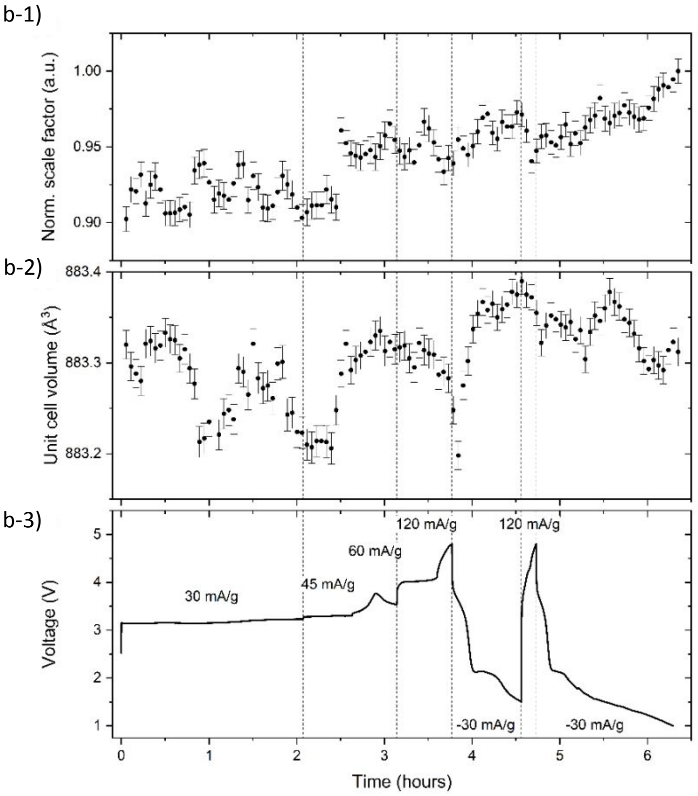

Figure S9. (a) *In operando* synchrotron XRD contour map (background subtracted and normalized) of the first two complete electrochemical cycles at 50 °C for $LiNi_{2/3}Nb_{1/3}O_2$ (x = 0.33). The corresponding electrochemical load curves (rate and voltage) are shown on the left side of the figure. The asterisks denote the reflections from the aluminium foil in the electrode. (b) b-1) Normalized scale factor as function of time. b-2) Unit cell volume as function of time. b-3) Potential as function of time.

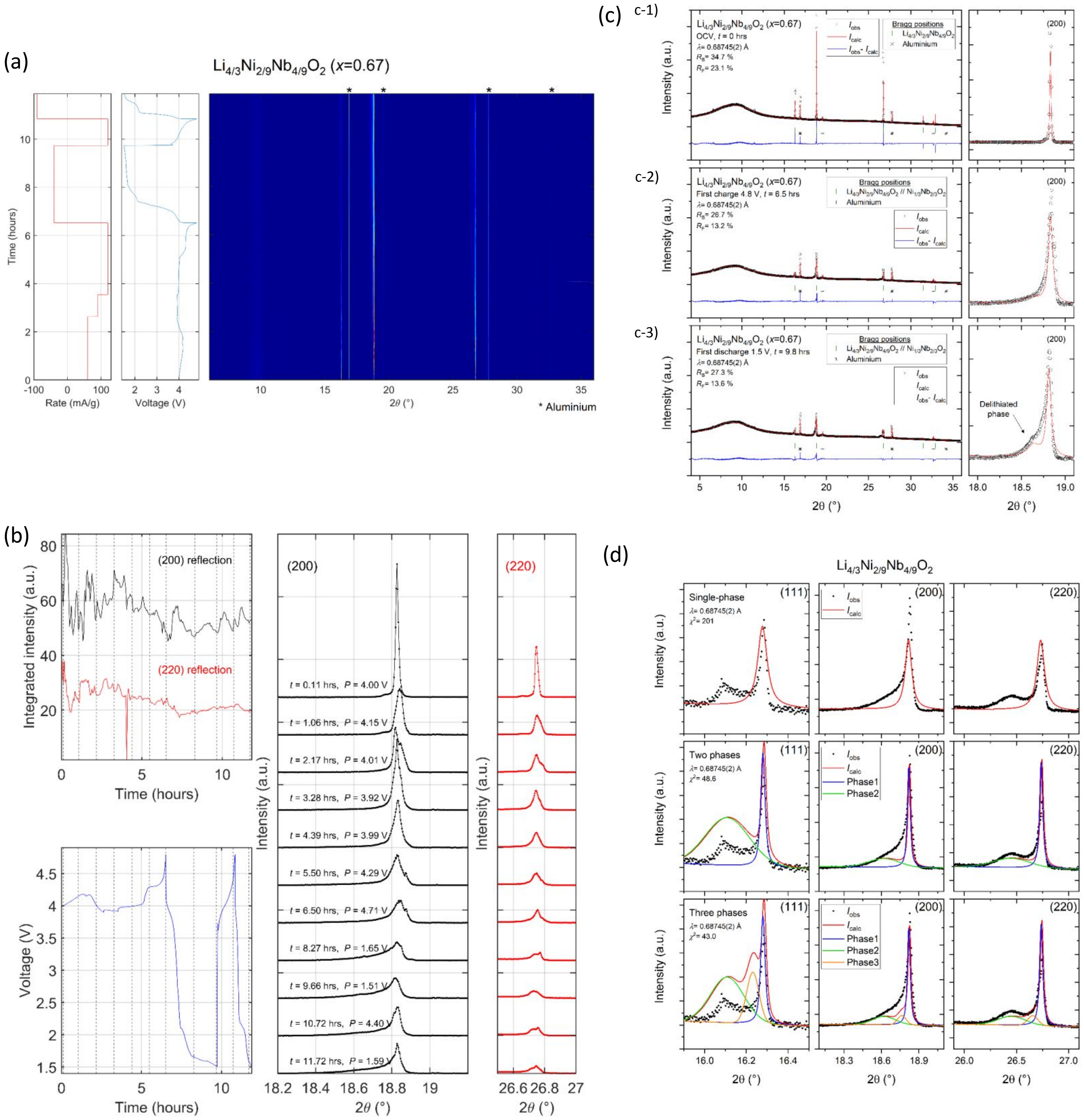

Figure S10. (a) *In operando* synchrotron XRD contour map (background subtracted and normalized) of the first two complete electrochemical cycles at 50 °C for $Li_{4/3}Ni_{2/9}Nb_{4/9}O_2$ (x = 0.67). The corresponding electrochemical load curves (rate and voltage) are shown on the left side of the figure. The asterisks denote the reflections from the aluminium foil in the electrode. (b) Evolution

of the (200) and (220) reflections during the two initial electrochemical cycles. The integrated intensities as function of time are plotted in the top left and the electrochemical load curves are plotted in the bottom left. The dashed lines indicate the times at which the patterns shown on the right were obtained. (c) Rietveld-refined fits with structural models to XRD data collected after c-1) prior to electrochemical cycling (OCV, 0 hrs), c-2) in the charged state (4.8 V, 6.5 hrs) and c-3) discharged state (1.5 V, 9.8 hrs). The patterns are plotted with the same absolute *y*-scale to illustrate the degradation of the main phase. An enhancement of the main (200) peak is shown on the right. The formation of the delithiated low crystallinity phase is observed from the appearance of a shoulder on the left side (larger cell) of the reflections as indicated by the arrow in c-3). (d) Enhancement of the (111), (200) and (220) reflections fitted by a single-, two-, and three rock salt phases.

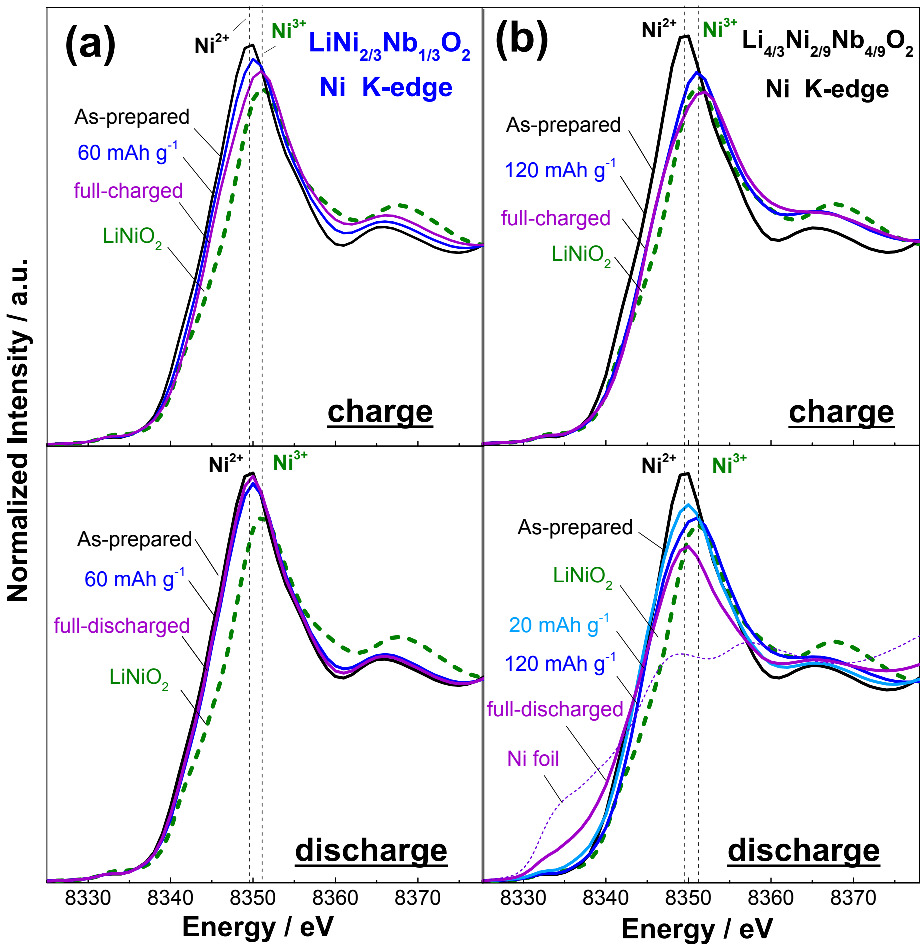

Figure S11 Change in the Ni K-edge XAS spectra of (a) $Li_{1-y}Ni_{2/3}Nb_{1/3}O_2$ (x = 0.33) and (b) $Li_{4/3-y}Ni_{2/9}Nb_{4/9}O_2$ (x = 0.67).

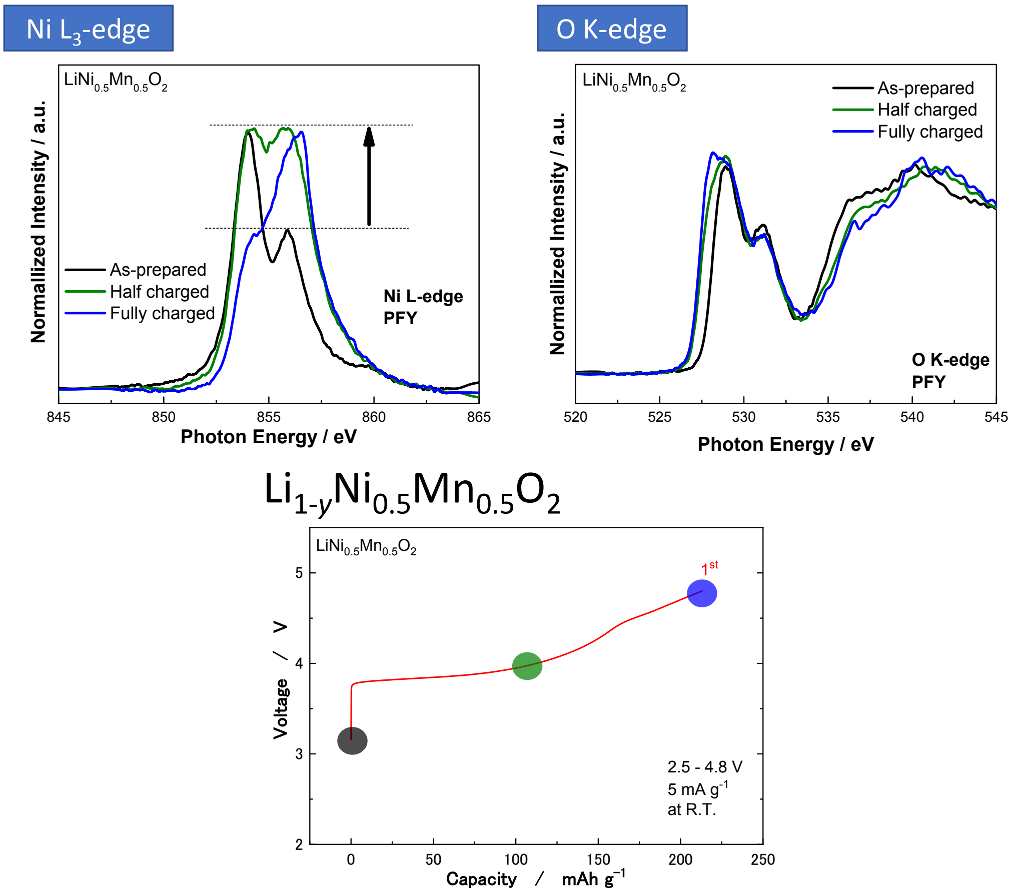

Figure S12. Changes in XAS spectra at Ni L-edge and O K-edge for layered $Li_{1-y}Ni_{0.5}Mn_{0.5}O_2$. A clear change in a Ni L-edge spectrum is observed for the half charged state (*ca*. 110 mA h g^{-1}) when compared with $Li_{1-y}Ni_{2/3}Nb_{1/3}O_2$. On further oxidation to 4.8 V, the formation of Ni^{4+} is noted, which is not evidenced for $Li_{1-y}Ni_{2/3}Nb_{1/3}O_2$. Peak shift of O K-edge to a lower energy region is also noted, and this observation is also clearly different from $Li_{1-y}Ni_{2/3}Nb_{1/3}O_2$.

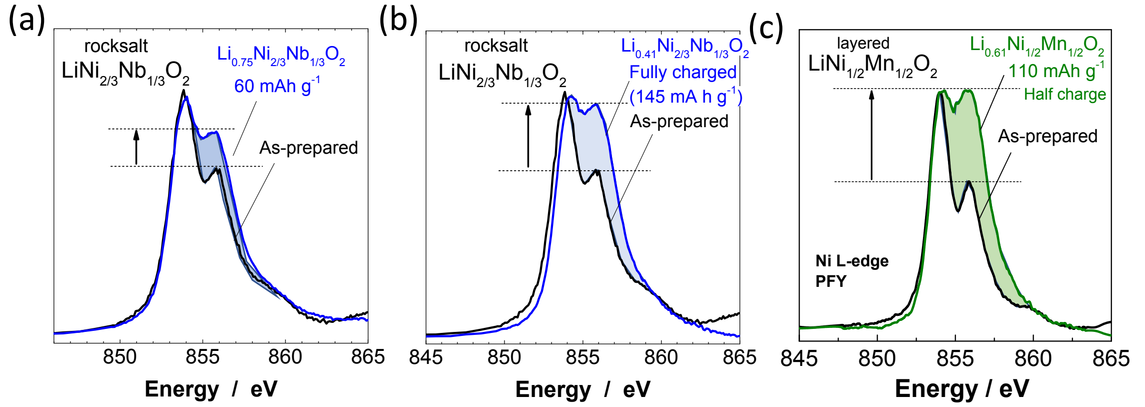

Figure S13. Comparison of normalized Ni L-edge XAS spectra for $Li_{1-y}Ni_{2/3}Nb_{1/3}O_2$ and $Li_{1-y}Ni_{1/2}Mn_{1/2}O_2$. From differences in the integrated peak area, the average oxidation state of Ni ions in fully charged $Li_{1-y}Ni_{2/3}Nb_{1/3}O_2$ (charged to 4.8 V) is expected to be lower than that of Ni ions in half charged $Li_{1-y}Ni_{1/2}Mn_{1/2}O_2$ because the change in integrated area before and after charge is relatively small for $Li_{1-y}Ni_{2/3}Nb_{1/3}O_2$. The estimated nominal oxidation state of Ni ions in half charged $Li_{1-y}Ni_{1/2}Mn_{1/2}O_2$ is calculated to be +2.78 on the basis of charge capacity (110 mA h g^{-1}).

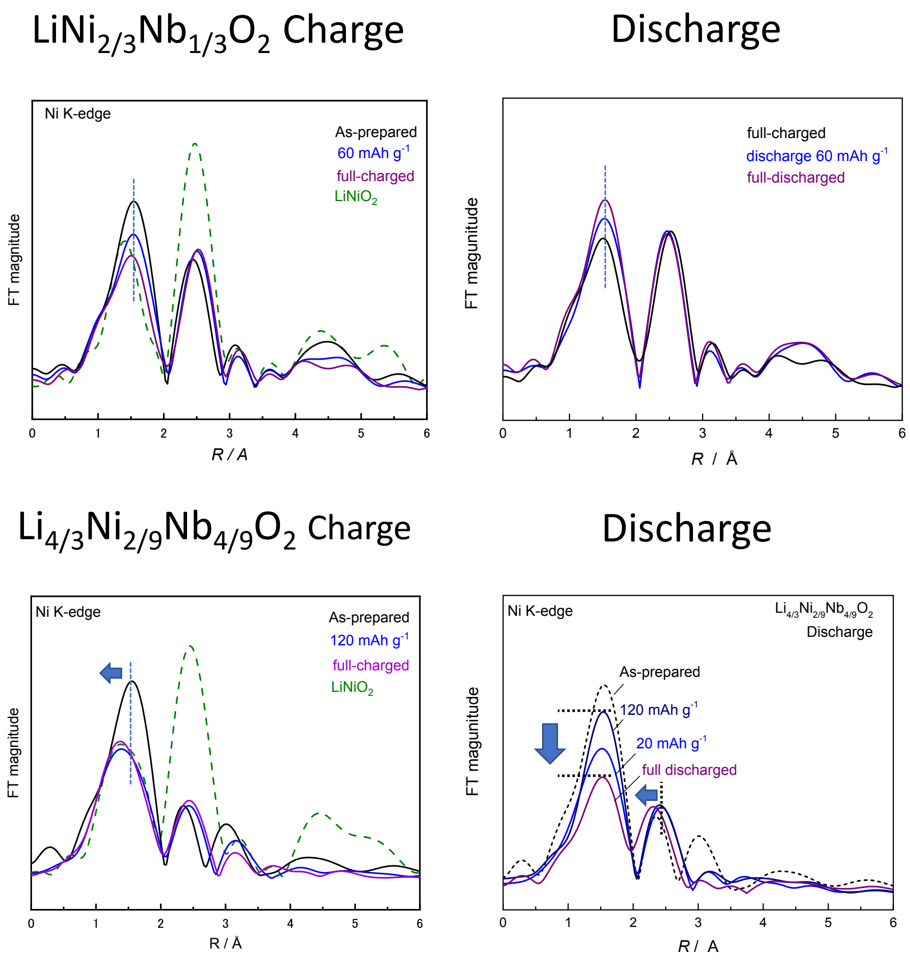

Figure S14. Extended X-ray absorption fine structure (EXAFS) spectra of stoichiometric $LiNi_{2/3}Nb_{1/3}O_2$ and Li-excess $Li_{4/3}Ni_{2/9}Nb_{4/9}O_2$ on charge/discharge processes. First and second coordination shells correspond to Ni-O and Ni-Ni pairs, respectively. Because the structures of both phases are classified as the disordered rocksalt structure for Ni ions, peak heights of second coordination shells are much lower compared with layered $LiNiO_2$. For the stoichiometric sample, distances of the Ni-O coordination are almost unchanged on charge, and this fact also supports the small contribution of Ni ions for charge compensation. Although the height of peaks is slightly

reduced on charge, this observation originates from the presence of the small amount of Ni^{3+}, which is known as a Jahn-Teller active ion. For the Li-excess system, after full discharge, the peak height of the first coordination shell is drastically reduced, and the interatomic distance for the second coordination clearly is shortened. This observation also supports the formation of nanosized and/or metallic nickel. An interatomic distance of Ni-Ni in metallic Ni is calculated to be 2.5 Å, which is shorter than Ni-Ni distances of *ca*. 2.9 Å for oxides.

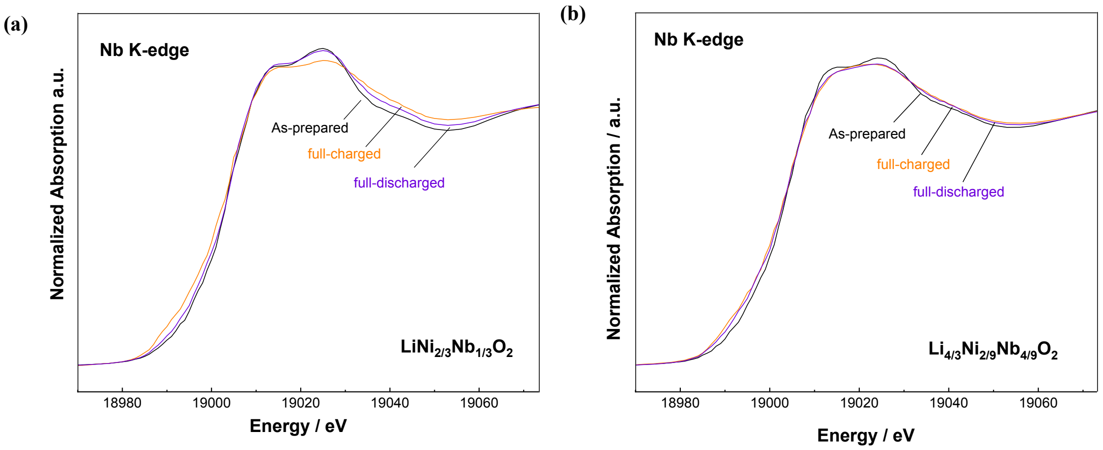

Figure S15. Nb K-edge XNAES spectra of $LiNi_{2/3}Nb_{1/3}O_2$ ($x = 0.33$) and $Li_{4/3}Ni_{2/9}Nb_{4/9}O_2$ ($x = 0.44$) electrodes during the 1st cycle. Only minor changes are observed in the profiles of spectra, which are expected to originate from local structure changes around Nb ions associated with Li extraction (for instance, K. Kubobuchi *et al*., *Journal of Applied Physics*, **120**, 142125, 2016). Therefore, the result indicates that Nb ions are not directly involved in charge compensation.

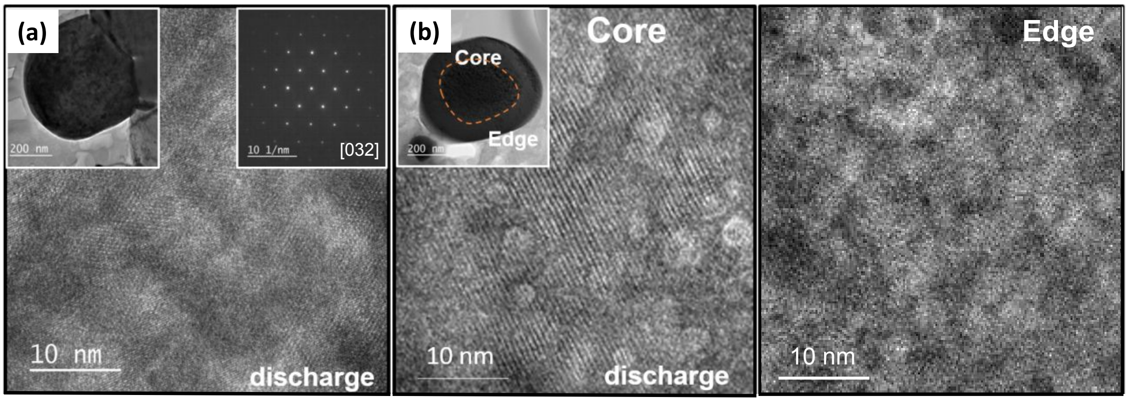

Figure S16. HR-TEM observation of electrochemically cycled samples; (a) $Li_{1-y}Ni_{2/3}Nb_{1/3}O_2$ and (b) $Li_{4/3-y}Ni_{2/9}Nb_{4/9}O_2$. The low-magnification TEM image and ED pattern in (a) are also shown. [032] zone axis for the stoichiometric sample corresponds to [111] for the parent rock salt phase. HR-TEM images collected for the core-region and edge-region of $Li_{4/3-y}Ni_{2/9}Nb_{4/9}O_2$ particles are shown in (b).

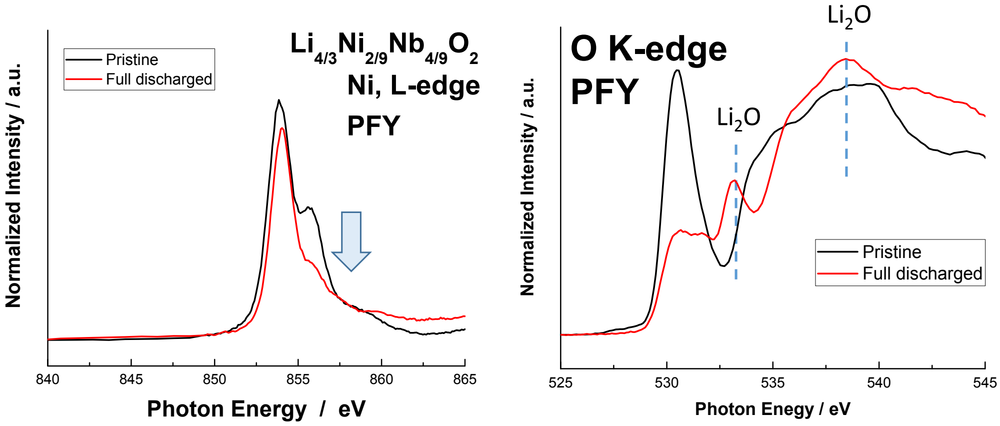

Figure S17. Ni L-edge and O K-edge spectra of $Li_{4/3-y}Ni_{2/9}Nb_{4/9}O_2$; comparison of the pristine sample to the fully discharged state at 1.5 V after charge to 4.8 V. Reduction of Ni ions is clearly

noted in the Ni L-edge XAS spectrum, and new peaks at 533.5 and 539 eV observed for the O K-edge spectrum would originate from the formation of nanosized Li_2O on discharge. O K-edge XAS spectra of Li_2O is found in literature (for instance, Qiao *et al.*, *PLOS ONE*, **7**, e49182, 2012). Note that a crystalline Ni metal phase was not found by XRD (**Figure S8**), suggesting that metallic Ni with an amorphous phase or that is nanosized is formed.

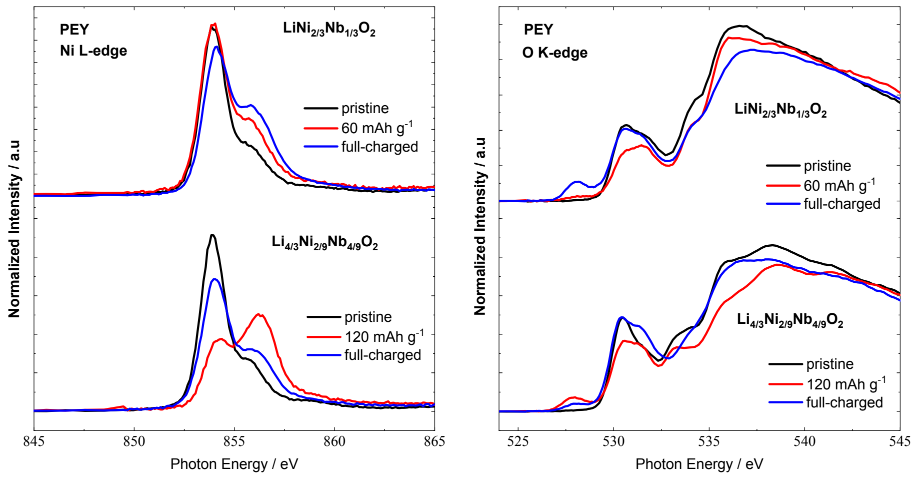

Figure S18. Ni L-edge and O K-edge XAS spectra of $LiNi_{2/3}Nb_{1/3}O_2$ ($x = 0.33$) and $Li_{4/3}Ni_{2/9}Nb_{4/9}O_2$ ($x = 0.67$) electrodes obtained at PEY mode which is surface sensitive. Holes are stabilized in the bulk of particles as observed in PFY mode (**Figure 4c**).

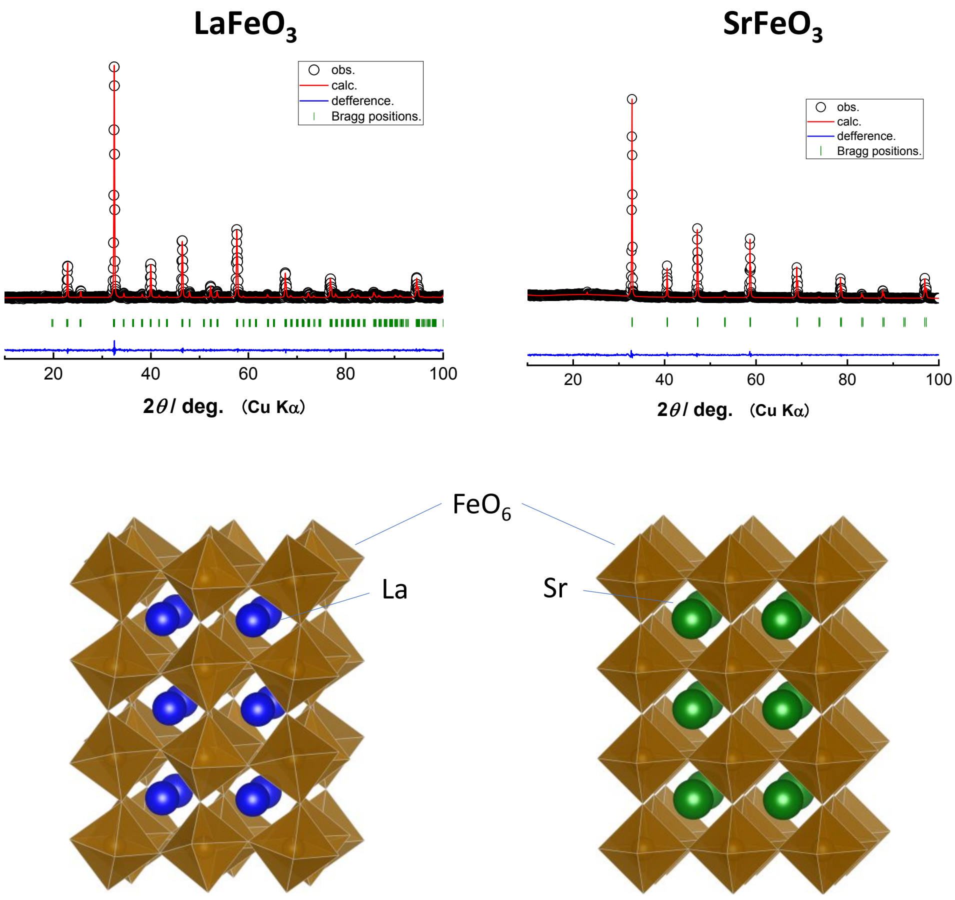

Figure S19. Results of Rietveld refinements for $SrFeO_3$ and $LaFeO_3$ used as reference materials for the XAS study. Schematic illustrations of crystal structures are also shown. Refined structural parameters are found in **Supporting Table S3** and **S4**.

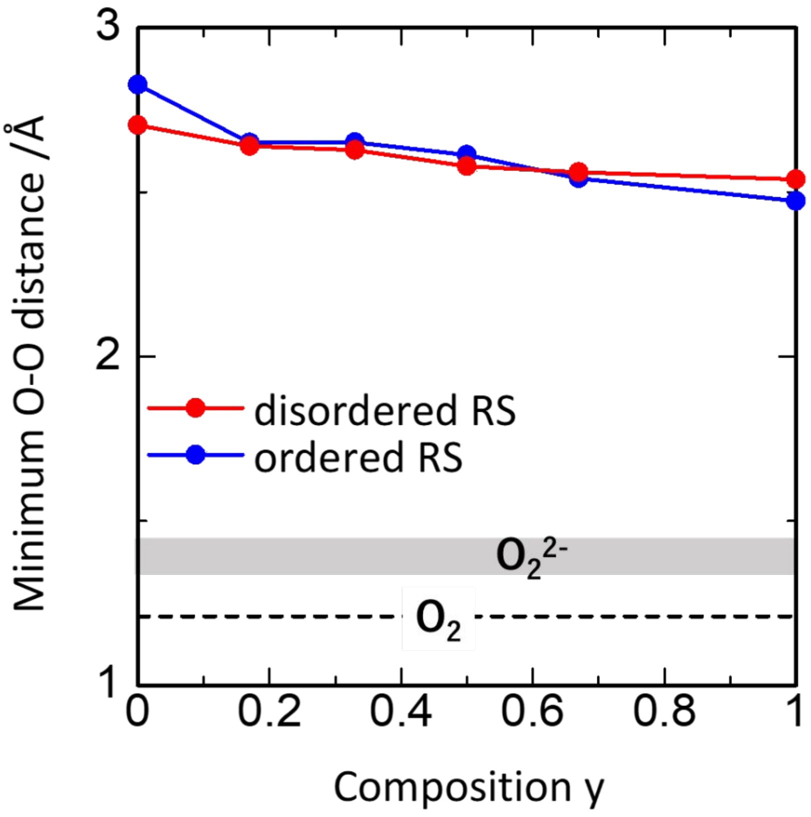

Figure S20. The minimum oxygen-oxygen interatomic distance in the disordered/ordered RS-type $Li_{1-y}Nb_{1/3}Ni_{2/3}O_2$. The O-O bond length for oxygen molecule, O_2 (1.21 Å), and peroxide ion, O_2^{2-} (~ 1.3 – 1.4 Å), is also shown for reference purposes.

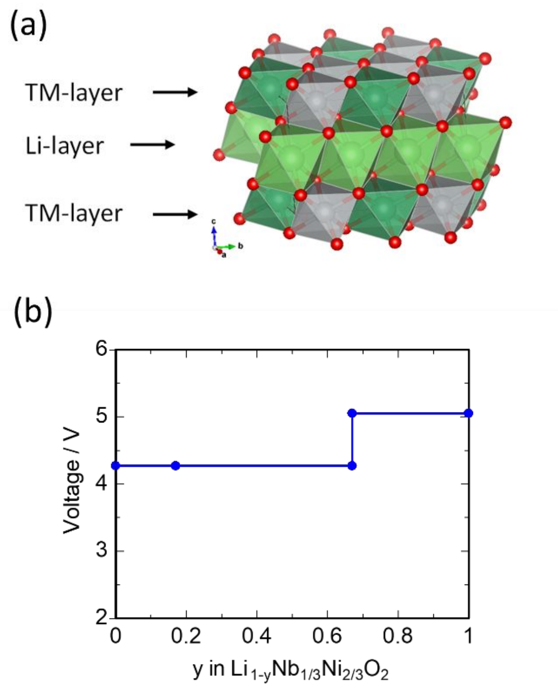

Figure S21. (a) A schematic illustration of the crystal structure of hypothetical layered-type $LiNb_{1/3}Ni_{2/3}O_2$ for first-principles calculations using the HSE06 functional. (b) Calculated voltages for electrochemical delithiation as a function of composition y in layered-type $Li_{1-y}Nb_{1/3}Ni_{2/3}O_2$.

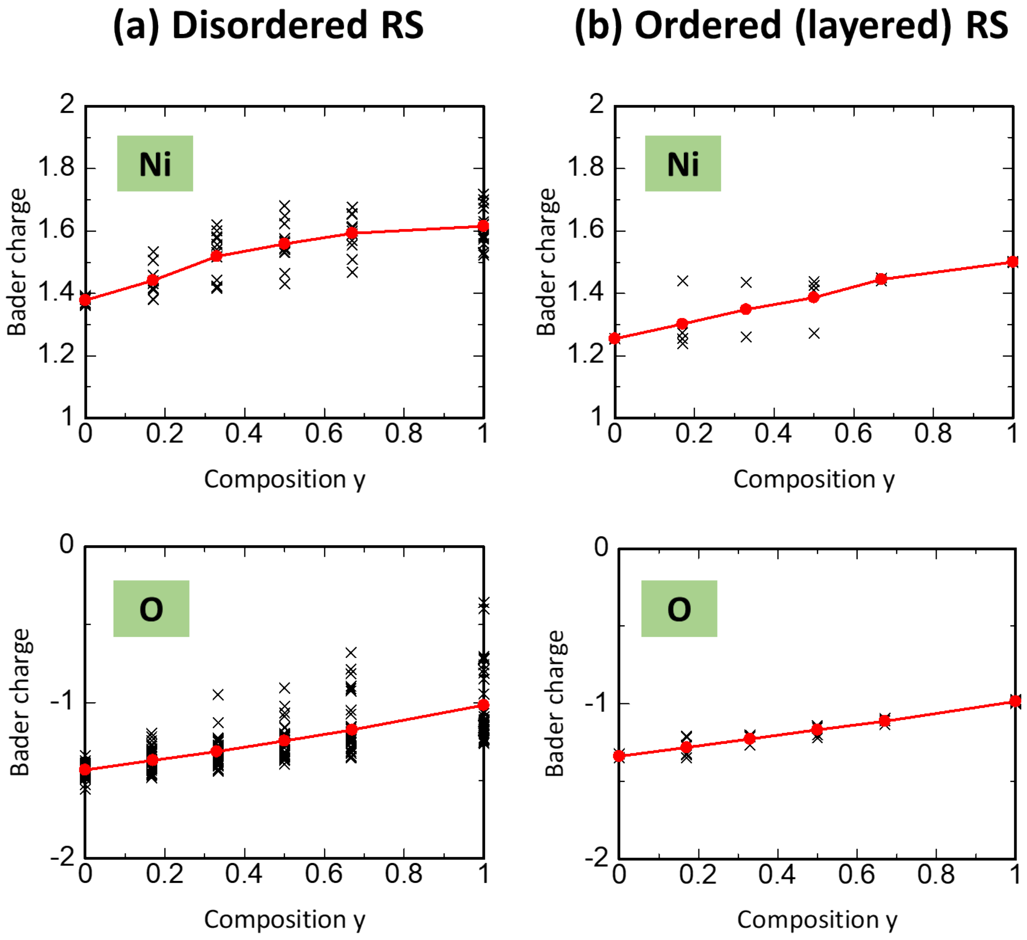

Figure S22. Changes in Bader charges for Ni and O ions as a function of composition *y* in the (a) disordered and (b) ordered RS-type $Li_{1-y}Nb_{1/3}Ni_{2/3}O_2$. Clear changes in Barder charge for O in the Ni-O-Ni configuration are noted for disordered $Li_{1-y}Nb_{1/3}Ni_{2/3}O_2$. For ordered $Li_{1-y}Nb_{1/3}Ni_{2/3}O_2$, the increase in Barder charge for O originates from the increase in covalency of Ni-O bonds by Ni oxidation on delithiation. Cross symbols represent Bader charges for individual ions, while red circles with a line correspond to averaged Bader charges.

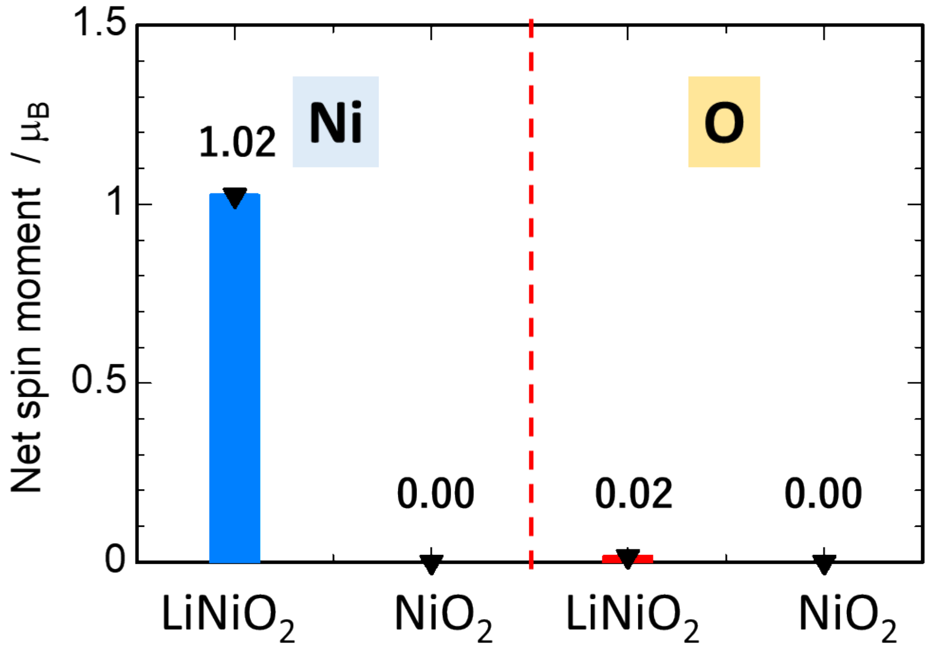

Figure S23. Comparison of calculated net spin moments for $LiNiO_2$ and □NiO_2. From the net spin moments, no evidence of hole creation is noted. A residual spin moment (0.07) for oxygen originates from large spin of Ni ions associated with covalency in Ni-O bonds.
